# Branched-chain amino acid metabolism controls membrane phospholipid structure in *Staphylococcus aureus*

**DOI:** 10.1016/j.jbc.2021.101255

**Published:** 2021-09-28

**Authors:** Matthew W. Frank, Sarah G. Whaley, Charles O. Rock

**Affiliations:** Department of Infectious Diseases, St Jude Children's Research Hospital, Memphis, Tennessee, USA

**Keywords:** fatty acid, fatty acid synthesis, *Staphylococcus aureus*, branched-chain amino acids, phospholipids, *a*C5-CoA, 2-methylbutyryl-CoA, ACP, acyl carrier protein, Bkd, branched-chain ketoacid dehydrogenase, C_2_-CoA, acetyl-CoA, FASII, type II fatty acid synthase, *h*, hydroxyl, *i*C4-CoA, isobutyryl-CoA, *i*C5-CoA, isovaleryl-CoA, MRM, multiple reaction monitoring, PG, phosphatidylglycerol, *t*, trans

## Abstract

Branched-chain amino acids (primarily isoleucine) are important regulators of virulence and are converted to precursor molecules used to initiate fatty acid synthesis in *Staphylococcus aureus*. Defining how bacteria control their membrane phospholipid composition is key to understanding their adaptation to different environments. Here, we used mass tracing experiments to show that extracellular isoleucine is preferentially metabolized by the branched-chain ketoacid dehydrogenase complex, in contrast to valine, which is not efficiently converted to isobutyryl-CoA. This selectivity creates a ratio of *anteiso*:*iso* C_5_-CoAs that matches the *anteiso*:*iso* ratio in membrane phospholipids, indicating indiscriminate utilization of these precursors by the initiation condensing enzyme FabH. Lipidomics analysis showed that removal of isoleucine and leucine from the medium led to the replacement of phospholipid molecular species containing *anteiso*/*iso* 17- and 19-carbon fatty acids with 18- and 20-carbon straight-chain fatty acids. This compositional change is driven by an increase in the acetyl-CoA:C_5_-CoA ratio, enhancing the utilization of acetyl-CoA by FabH. The acyl carrier protein (ACP) pool normally consists of odd carbon acyl-ACP intermediates, but when branched-chain amino acids are absent from the environment, there was a large increase in even carbon acyl-ACP pathway intermediates. The high substrate selectivity of PlsC ensures that, in the presence or the absence of extracellular Ile/Leu, the 2-position is occupied by a branched-chain 15-carbon fatty acid. These metabolomic measurements show how the metabolism of isoleucine and leucine, rather than the selectivity of FabH, control the structure of membrane phospholipids.

Fatty acid synthesis is a vital aspect of bacterial metabolism and carried out by a series of reaction steps catalyzed by individual proteins known as the type II fatty acid synthase (FASII) (1). Fatty acids are the major building blocks of the phospholipids, and the biophysical properties of cellular membranes are controlled in large part by the composition of the acyl chains (2). Bacteria alter the membrane fatty acid composition to modify membrane fluidity in response to the environment using two different biochemical schemes (Fig. 1). In model organisms like *Escherichia coli* and *Streptococcus pneumoniae*, membrane fluidity is altered by varying the saturated:unsaturated fatty acid ratio at a branch point within FASII (Fig. 1*A*) The enzymatic mechanisms and transcription factors that regulate unsaturated fatty acid synthesis are known in these systems (2, 3, 4, 5, 6, 7, 8, 9, 10, 11). Unsaturated fatty acid synthesis arises, and the expression levels/activities of 3-hydroxyacyl acyl carrier protein (ACP) dehydratase/isomerase (FabA) and 3-ketoacyl-ACP synthase I (FabB) determine the amount of unsaturated fatty acid produced (12). Many Gram-positive bacteria do not produce any unsaturated fatty acid, and the ratio of branched-chain to straight-chain fatty acids is a major determinant of membrane fluidity (2, 13, 14, 15, 16, 17). Experiments with model membranes show that phospholipids with branched-chain fatty acids are more fluid than their straight-chain homologs resulting in reduced lipid bilayer thickness and an increase in membrane bilayer fluidity (18, 19). Fatty acids with *anteiso* branching are more effective at fluidizing the membrane than fatty acid with *iso* branching. There is a correlation between an increase in the proportion of *anteiso* fatty acids as an adaptive response to reduced temperatures that is especially important for the survival of cold tolerant pathogens like *Listeria monocytogenes* (16, 20, 21). Unlike unsaturated fatty acid synthesis, the branched chain is introduced at the FabH step, the initial condensation reaction of FASII (Fig. 1*B*). Finally, some bacteria, like *Bacillus* and *Pseudomonas*, possess oxidative desaturases that introduce double bonds into existing fatty acids (22, 23).

**Figure 1:**
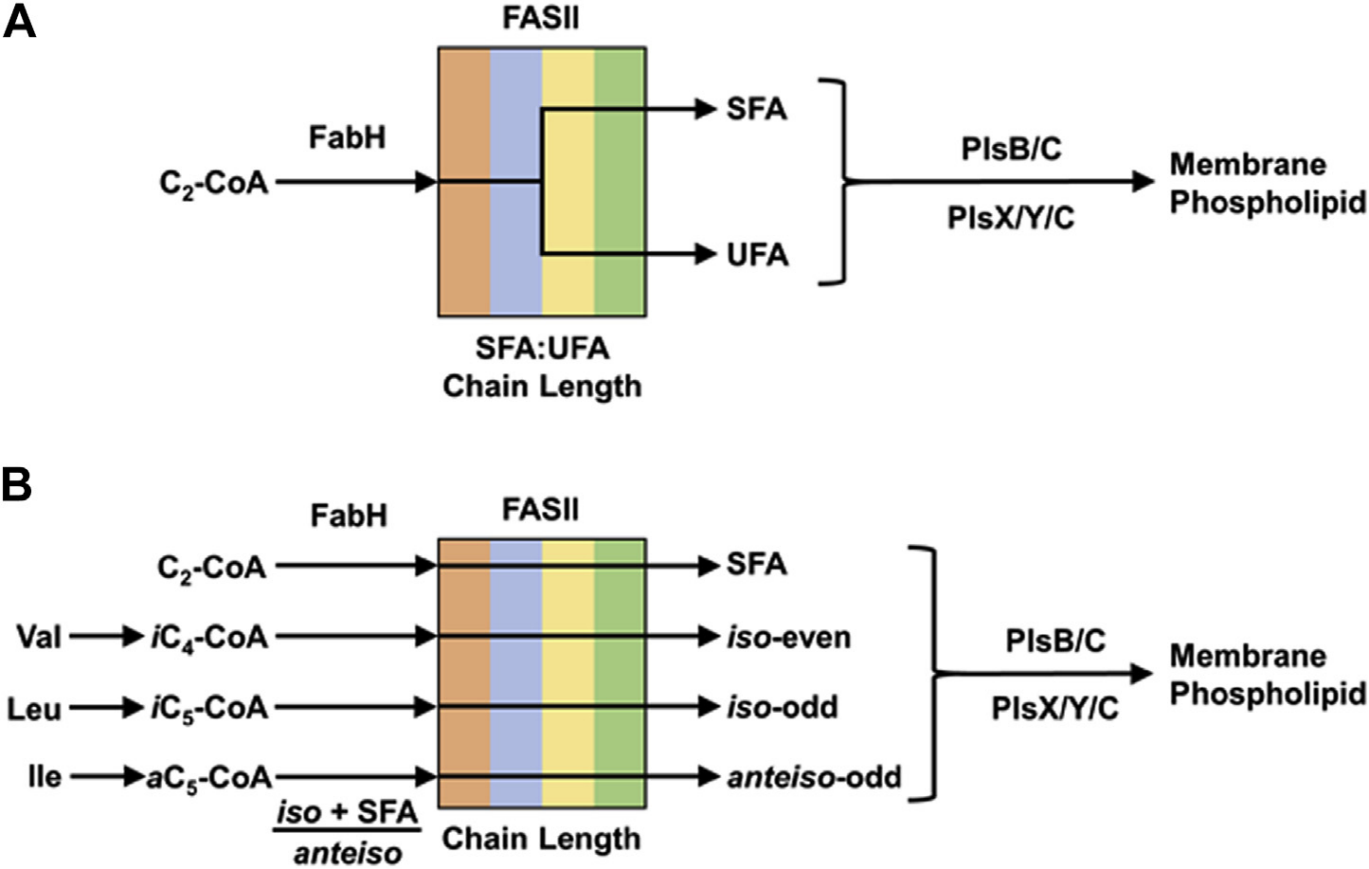
**Two different schemes used by bacteria to control fatty acid structure and membrane biophysical properties.***A*, many bacteria have FASII elongation systems that produce saturated fatty acid (SFA) and unsaturated fatty acid (UFA) straight-chain fatty acids. These organisms control their membrane biophysical properties by varying the proportion of UFA at a branch point in the middle of the elongation cycle. The genetic and biochemical mechanisms that regulate branch points within FASII are best known in *Escherichia coli* (1, 2), which is diagrammed here. However, organisms that make straight-chain fatty acids and use the PlsX/Y/C pathway, like *Streptococcus pneumoniae*, also control composition using a genetically controlled branch point in the middle of FASII (10). *B*, many bacteria do not make UFA and instead produce predominantly *iso* and *anteiso* branched-chain fatty acids. These organisms alter the proportions of branched-chain fatty acids to modify their membrane biophysical properties (1, 2). The proportion of straight *iso* and *anteiso* fatty acids are determined at the initiation step (FabH). In both schemes, the activities of the elongation condensing enzymes and acyltransferases control fatty acid chain length. FASII, type II fatty acid synthase.

Branched-chain amino acids (Ile, Leu, and Val) are precursors to the acyl-CoA primers used by FabH to initiate FASII in the Gram-positive pathogen *Staphylococcus aureus*. The major fatty acids are derived from Ile based on the predominance of *anteiso*-branched-chain fatty acids present in the phospholipids (24). Extracellular amino acids are diverted to FASII by their conversion to their respective ketoacids by the IlvE transaminase (13, 20, 25). The ketoacids are decarboxylated by the branched-chain ketoacid dehydrogenase (Bkd) complex to form the respective acyl-CoAs (26). Val gives rise to isobutyryl-CoA (*i*C_4_-CoA), Leu is converted to isovaleryl-CoA (*i*C_5_-CoA), and Ile becomes 2-methyl-butyryl-CoA (*a*C_5_-CoA). Initiation with an amino acid–derived acyl-CoA results in branched-chain fatty acid synthesis, whereas initiation with acetyl-CoA (C_2_-CoA) gives rise to straight-chain fatty acids. In *S. aureus*, there is a single 3-ketoacyl-ACP synthase III (FabH) that draws from the short-chain acyl-CoA primer pool to initiate FASII (27, 28). Purified FabH has a distinct preference for C_5_-CoA compared with C_2_-CoA but does not have a high selectivity for either *i*C_5_-CoA or *a*C_5_-CoA (13, 27, 28), making it difficult to understand how FabH can account for the preferential use of *a*C_5_-CoA to prime FASII or the modulation of fatty acid composition in response to environmental conditions. Changes in media composition and amino acid transporter activities (14, 15) significantly alter fatty acid composition and are consistent with control being exerted by the supply rather than the utilization of FabH substrates. How amino acid metabolism is connected to FASII has not been adequately explored.

The goal of this work is to determine how branched-chain amino acid metabolism impacts the acyl-CoA and acyl-ACP intermediate pool compositions and membrane phospholipid structure in *S. aureus*. Isotopic tracer experiments with mass-tagged branched-chain amino acids show that the Bkd pathway produced a FabH precursor pool that reflects the *anteiso*:*iso* ratio found in phospholipids. Val metabolism to *i*C_4_-CoA is minimal. The removal of Ile elevates phosphatidylglycerol (PG) molecular species containing 18- and 20-carbon straight-chain fatty acids. The ACP pool normally consists of odd carbon acyl-ACP and hydroxyacyl-ACP intermediates. Ile limitation decreases the 2-methylbutyryl-CoA pool and increases the level of even carbon acyl-ACP FASII intermediates initiated from the utilization of C_2_-CoA. These metabolomic measurements show how the nutritional state with respect to Ile and Leu regulates the synthesis of straight-chain fatty acid for the 1-position acyl chain, whereas 15-carbon branched fatty acids remain the dominant 2-position acyl chain.

## Results

### Branched-chain amino acid utilization

**Figure 2:**
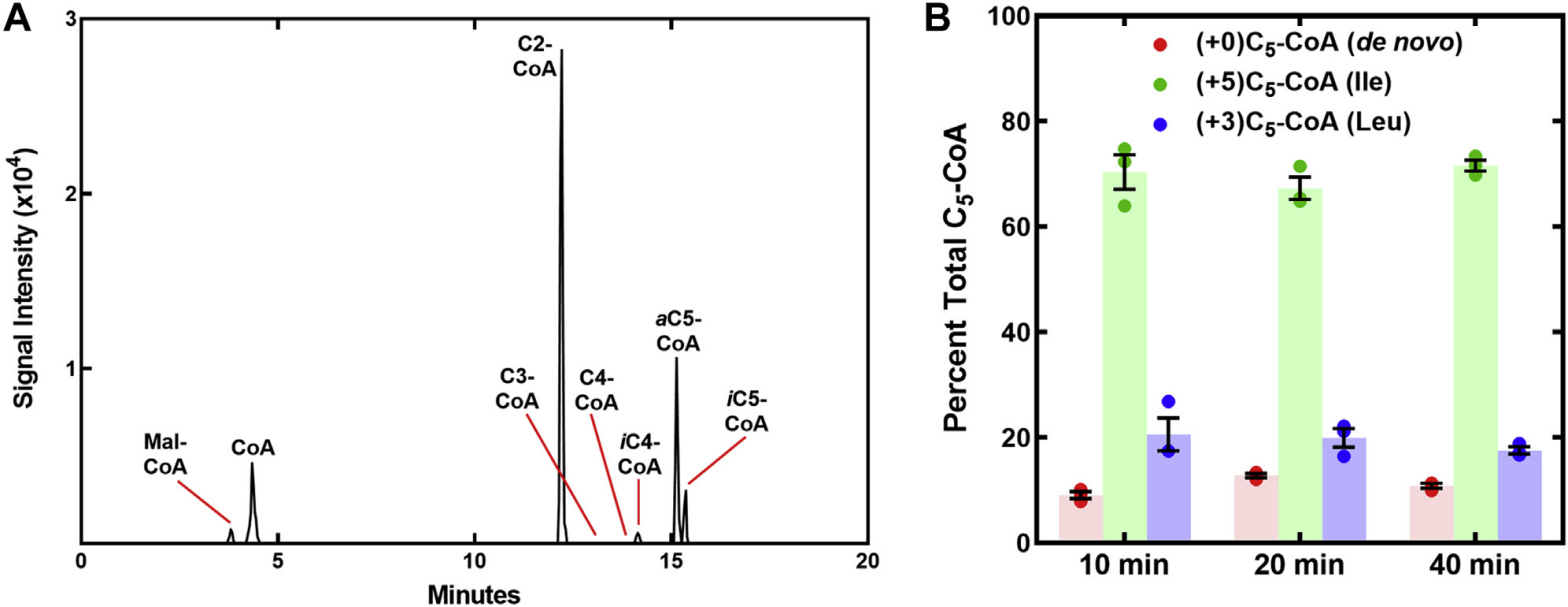
**Acyl-CoA pools in *Staphylococcus aureus*.***A*, MS profiling of the acyl-CoA pool of *S. aureus* strain AH1263 grown in defined medium containing Ile, Leu, and Val. The identities of the peaks are indicated along with the elution positions of other acyl-CoAs that were below the detection level in this experiment condition. *B*, metabolic origin of the C_5_-CoA pool in *S. aureus* strain AH1263 (wildtype) labeled in defined medium containing the heavy branched-chain amino acids [^13^C_6_]Ile, [*d*_3_]Leu, and [^13^C_5_,^15^N]Val in place of their normal counterparts. Triplicate biological replicates were obtained at the indicated time points, and the contributions of Ile (+5)*a*C_5_-CoA and Leu (+3)*i*C_5_-CoA to the total C_5_-CoA pool were determined by MS, and the means ± SEM are plotted. (+4)*i*C_4_-CoA derived from [^13^C_5_,^15^N]Val was present in trace quantities.

The impact of branched-chain amino acids of the intermediate metabolite pools that support FASII was examined in wildtype *S. aureus* strain AH1263 (29) grown in defined medium as described under the *Experimental procedures* section. The acyl-CoA precursors available to FabH in *S. aureus* were analyzed by LC–MS/MS of the extracted CoA thioester pool, and the CoA thioester content was normalized by the inclusion of an [^13^C_2_] C_2_-CoA internal standard in each replicate. A representative scan of *S. aureus* grown in defined media shows that C_2_-CoA is the major CoA thioester (Fig. 2*A*). The *i*C_5_-CoA and *a*C_5_-CoA species are resolved by chromatography and are the next most abundant thioesters. The *a*C_5_-CoA:*i*C_5_-CoA ratio was 3.5 illustrating that the precursor for *anteiso* fatty acids is the most abundant branched-chain primer available. CoA is a significant component, and low levels of malonyl-CoA and *i*C_4_-CoA are detected. Butyryl-CoA and propionyl-CoA are not detected when grown in defined growth medium.

The utilization of amino acids for membrane synthesis was traced using defined growth media with Ile, Leu, and Val substituted with [^13^C_6_]Ile, [*d*_3_]Leu, and [^13^C_5_,^15^N]Val to introduce specific mass tags that allow the origin of each fatty acid produced to be traced to its amino acid source. *a*C_5_-CoA derived from [^13^C_6_]Ile will have a +5 mass, *i*C_5_-CoA derived from [*d*_3_]Leu will have a +3 mass, and *i*C_4_-CoA derived from [^13^C_5_,^15^N]Val will have a +4 mass. Wildtype strain AH1263 was grown to midlog phase in defined medium, and switched to media containing the heavy amino acids and samples removed at 10, 20, and 40 min and analyzed by LC–MS/MS. Samples obtained at the 10-min time point show that (+5)C_5_-CoA derived from [^13^C_6_]Ile is the major species with (+3)C_5_-CoA derived from [*d*_3_]Leu being less abundant (Fig. 2*B*). The ratio of (+5)C_5_-CoA:(+3)C_5_-CoA was 3.5. Approximately 10% of the C_5_-CoA pool was unlabeled corresponding to the contribution of 2-methylbutyrate and isovalerate arising from *de novo* Ile and Leu biosynthesis. We only detected a trace of (+4)*i*C_4_-CoA in our analysis consistent with the low abundance of *i*C_4_-CoA in the CoA thioester pool (Fig. 2*A*). The contributions of Ile and Leu to the C_5_-CoA pool remained constant over the 40-min labeling period showing that the system reached a steady state within 10 min. These data show that growth in rich media typically used in the laboratory (like LB), the major C_5_-CoA precursor available to FabH is derived from extracellular Ile. Because the concentrations of Ile and Leu in the defined medium are both 50 μg/ml, these data mean that extracellular Ile is selectively metabolized by the Bkd pathway compared with Leu. Val is not a significant fatty acid precursor.

**Figure 3:**
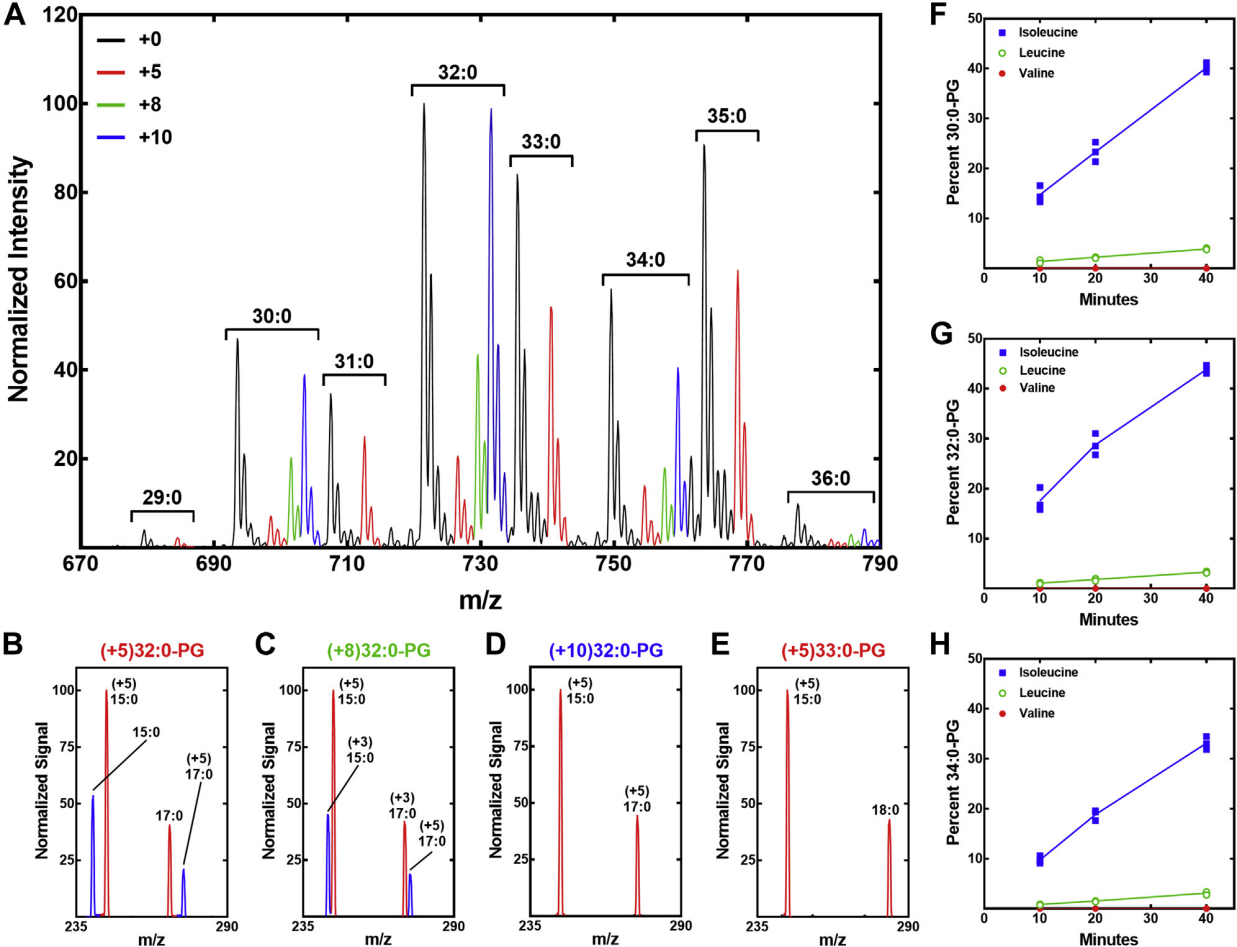
**Utilization of branched-chain amino acid precursors for phospholipid synthesis.***A*, a representative mass spectrum of PG molecular species at the 20-min time point following isotopic labeling with [^13^C_6_]Ile, [*d*_3_]Leu, and [^13^C_5_,^15^N]Val. The individual molecular species are labeled above the brackets with the isotopically labeled peaks color coded according to their increased mass. Pre-existing and unlabeled peaks are *black*, the +5 peaks derived from [^13^C_6_]Ile are *red*, the +8 peaks derived from [^13^C_6_]Ile and [*d*_3_]Leu are *green*, and the +10 peaks derived from [^13^C_6_]Ile are *blue*. The individual peaks were fragmented to determine the fatty acid composition. *B*, acyl chain composition of the (+5)32:0-PG peak. *C*, acyl chain composition of (+8)32:0-PG peak. *D*, acyl chain composition of (+10)32:0-PG peak. *E*, acyl chain composition of (+5)33:0-PG peak. Rates of Ile, Leu, and Val incorporation into the individual PG molecular species were obtained from three biological replicates, and the percentage contribution of each of the three amino acids to the total ion current in three of the prominent PG molecular species was calculated using LipidView software (Sciex). *F*, 30:0-PG. *G*, 32:0-PG. *H*, 34:0-PG. PG, phosphatidylglycerol.

The flow of branched-chain amino acids into membrane phospholipids was assessed by determining the isotopic distribution in the PG molecular species over the time course. The correspondence between the MS peaks and the PG structures is shown in Figure S1. A sample mass spectrum obtained at the 20-min time point illustrates the primary data and how it was analyzed (Fig. 3*A*). The even and odd numbered PG molecular species had distinctly different labeling patterns. For example, three new peaks (+5, +8, and +10) appeared in the 32:0-PG (17:0/15:0) peak cluster. The (+5)32:0-PG peak corresponded to one *anteiso*-fatty acid derived from [^13^C_6_]Ile combined with another branched-chain fatty acid from the unlabeled C_5_-CoA precursor pool. Analysis of the fragmentation pattern of this peak showed that it was predominately 17:0/(+5)*a*15:0, although (+5)*a*17:0/15:0-PG was also present (Fig. 3*B*). The (+8)32:0-PG peak corresponded to one *anteiso*-fatty acid arising from Ile combined with an *iso*-fatty acid arising from Leu. Fragmentation of this peak showed that (+3)*i*17:0/(+5)*a*15:0-PG was the major species (Fig. 3*C*). The most abundant new peak in the 32:0-PG cluster had a +10 mass (*m/z* = 731), meaning that it contained two *anteiso*-fatty acids derived from Ile. Fragmentation of this peak confirmed it was composed of (+5)*a*17:0/(+5)*a*15:0-PG (Fig. 3*D*). This same ratio of +5, +8, and +10 molecular species was found in all even numbered PG species (30:0, 32:0, and 34:0) (Fig. 3*A*). Only a +5 peak was detected in the odd-numbered molecular species (29:0, 31:0, 33:0, and 35:0) corresponding to one unlabeled even-numbered fatty acid paired with an *a*15:0 derived from [^13^C_6_]Ile. Fragmentation of the (+5)33:0-PG confirmed that it was 18:0/(+5)*a*15:0-PG (Fig. 3*E*). We did not detect +4 peaks in the odd-numbered PG molecular species, meaning that Val is not the precursor to the even-carbon acyl chains. Rather, these even-number fatty acids are not labeled indicating they arose from C_2_-CoA, the only available even-chain primer in the CoA thioester pool (Fig. 2*A*). We did not detect +3 peaks in the even-numbered PG molecular species, meaning that the straight-chain fatty acids are paired with *a*15:0 derived from Ile and not paired with *i*15:0 from Leu. The >2.2-fold higher signal of 15:0 in the fragmentation patterns of all PG molecular species indicates that 15:0 occupies the 2-position (30, 31, 32) consistent with previous analytical data showing that 15:0 almost exclusively occupies the 2-position of *S. aureus* phospholipids (24, 33, 34). The percentage of each heavy molecular species calculated from triplicate biological replicates illustrates the linear flow of amino acids into each molecular species over the time course (Fig. 3, *F*–*H*).

### Isoleucine limitation and PG structure

**Figure 4:**
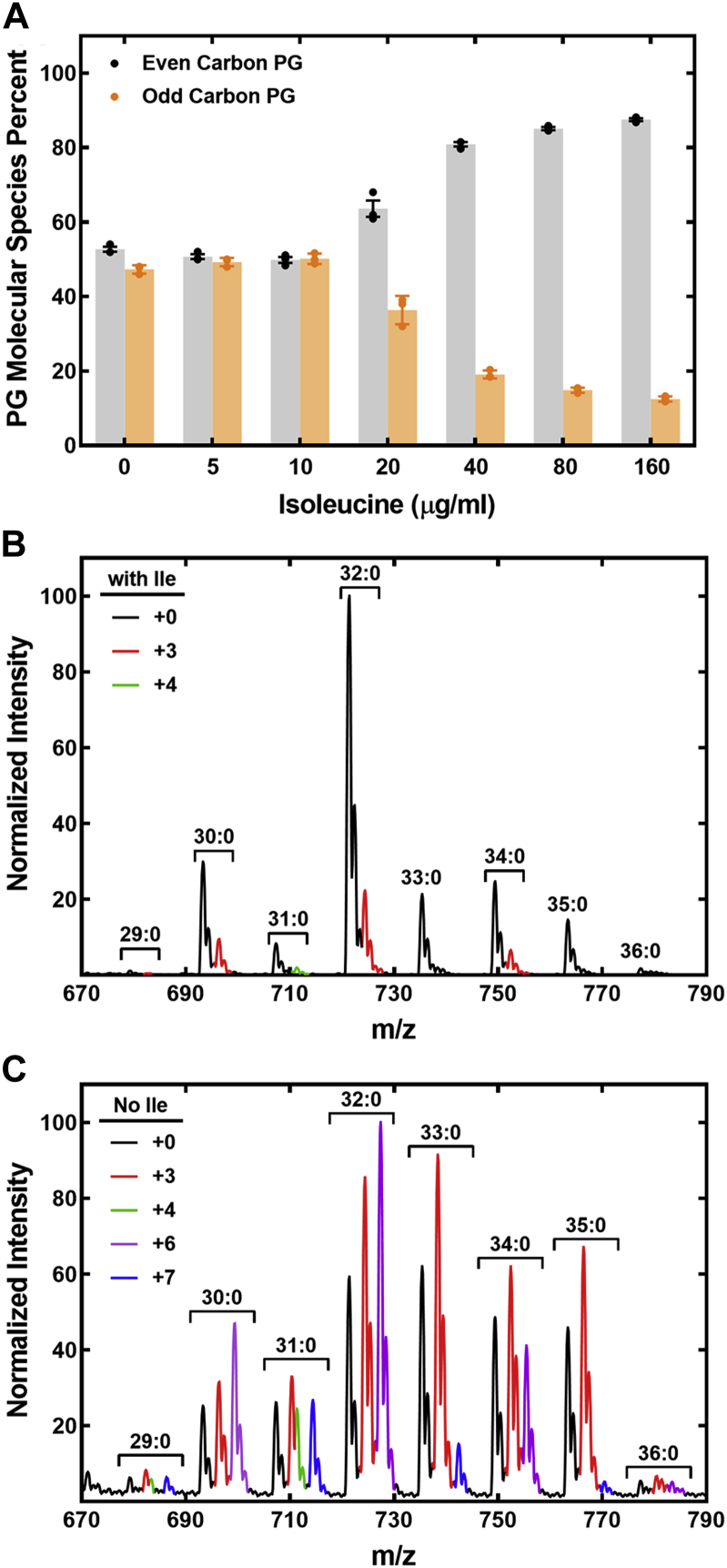
**Ile regulation of PG molecular species composition.***A*, *Staphylococcus aureus* strain AH1263 was grown in defined medium containing the indicated concentrations of Ile. The percent of the ion current attributed to even carbon PG (two odd branched-chain fatty acids) and odd carbon (straight + odd carbon fatty acid) was calculated at each Ile concentration. The data are plotted as mean ± SEM with three biological replicates. *B*, representative PG molecular species profile of strain AH1263 grown in defined medium containing Ile plus [*d*_3_]Leu and [^13^C_5_,^15^N]Val. *Red* peaks indicate Leu incorporation (+3), and *green* peaks indicate Val labeling (+4). *C*, representative PG molecular species profile of strain AH1263 grown in defined medium lacking Ile plus [*d*_3_]Leu and [^13^C_5_,^15^N]Val. The isotopic differences in each cluster of PG molecular species are color coded as shown in the inset key. PG, phosphatidylglycerol.

The growth medium has a significant impact on the fatty acid composition of *S. aureus* (14, 35), and striking changes are observed in the membrane PG molecular species in *S. aureus* in infection models (36, 37). In the thigh infection model, even-chain fatty acids are paired with 15:0 predominated, and this effect could be created in the laboratory by removing Ile from the growth medium (36). However, these experiments did not address whether even carbon fatty acids were straight chain or *iso* branched (Val derived) or whether the odd carbon fatty acids are *iso* (Leu derived) or *anteiso* (Ile derived). Our baseline experiments validated these prior results (36) showing that the removal of Ile from the defined growth media resulted in a marked increase in PG molecular species containing even-chain fatty acid (Fig. S2). At Ile concentrations ≥40 μg/ml, even carbon molecular species predominated, whereas at Ile concentrations ≤10 μg/ml, odd and even carbon PG molecular species were equally abundant (Fig. 4*A*). Each of the odd PG molecular species decreased, and each of the even carbon species increased as Ile increased in the medium (Fig. S3).

Isotopic labeling was performed to determine how the absence of Ile from the medium alters the incorporation of Leu and Val into membrane phospholipids. Strain AH1263 was labeled with [*d*_3_]Leu and [^13^C_5_,^15^N]Val in the presence and absence of unlabeled Ile. Mass spectra in the presence of Ile showed detectable incorporation of Leu (+3) into all even carbon number PG molecular species. Val incorporation (+4) was only detected as a minor component of the 31:0-PG cluster (Fig. 4*B*). In the absence of Ile, the incorporation of Leu, and to a lesser extent Val, into PG was significantly enhanced (Fig. 4*C*). The even carbon molecular species (30:0, 32:0, and 34:0) had distinct +3 peaks indicating pairing of an *iso* fatty acid with an unlabeled *iso* or *anteiso* fatty acid. These even carbon number molecular species also had significant +6 peaks showing that *iso* fatty acids derived from Leu are incorporated into both positions of the glycerol backbone. The 29:0 and 31:0 PG species had detectable +4 peaks indicating the corporation of Val into these species as an even-carbon acyl chain. These Val-derived fatty acids are also paired with an *iso* fatty acid derived from Leu giving rise to a +7 peak in both cases. The 33:0 and 35:0 PG molecular species that are elevated in the absence of Ile (Fig. S2, *C* and *D*) have only +3 counterparts, meaning that these molecular species consist of an even-chain fatty acid that is not derived from amino acid metabolism and an *iso* fatty acid derived from Leu. These data illustrate the large impact of Ile availability on membrane phospholipid structure.

**Figure 5:**
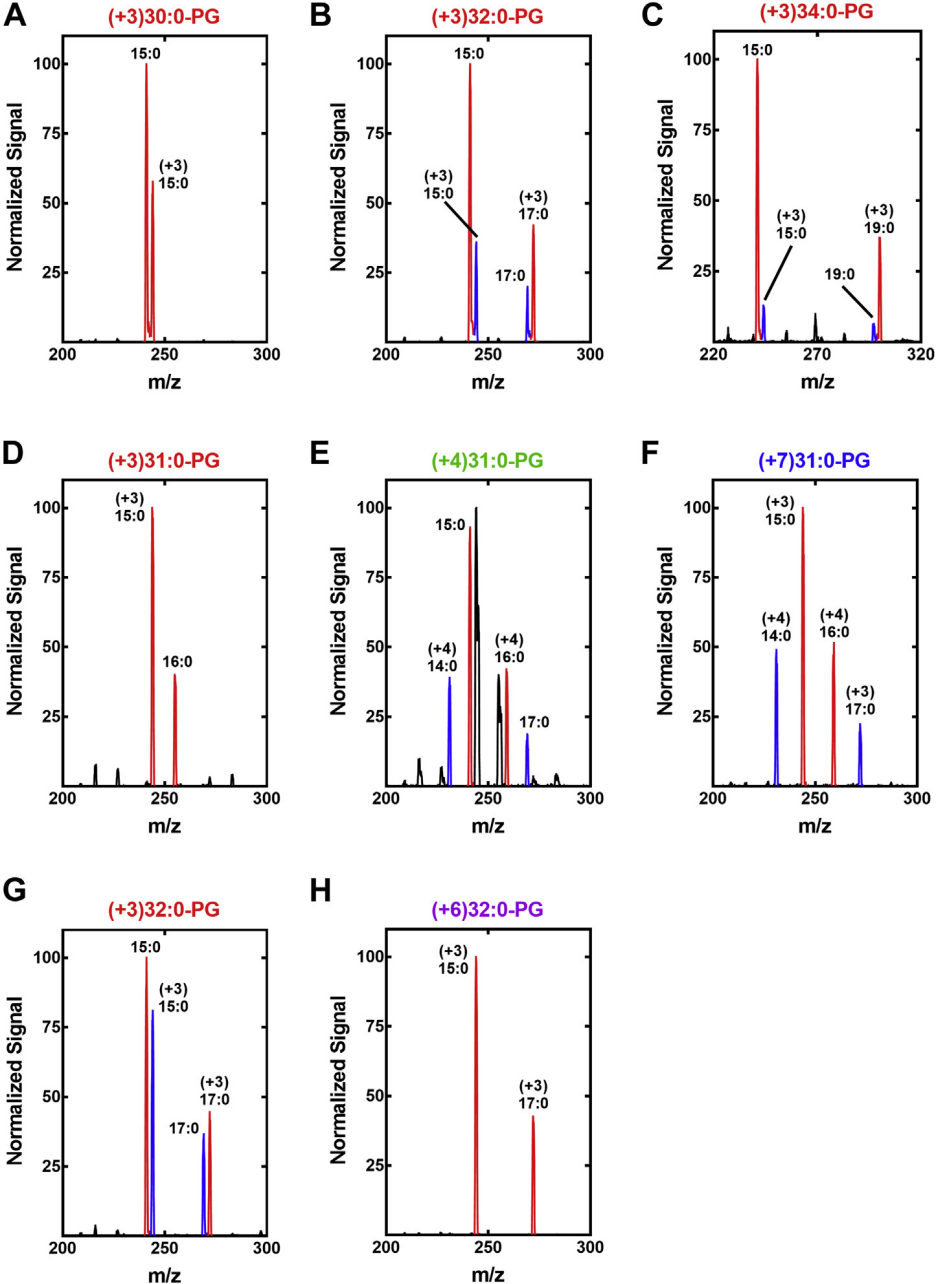
**Acyl chain compositions of PG molecular species in media lacking Ile.** Strain AH1263 was grown in defined medium with (panels *A*–*C*) or without (panels *D*–*H*) Ile and containing [*d*_3_]Leu and [^13^C_5_,^15^N]Val. Fatty acids with +3 mass are derived from Leu, and those with a +4 mass are derived from Val, and the individual PG molecular species were fragmented to identify the constituent isotopically labeled fatty acid. Panels *A*–*C* analyze the +3 peaks from Figure 4*B*. *A*, the (+3)30:0-PG peak consisted of (+3)15:0/15:0-PG (*red*). *B*, the (+3)32:0-PG peak consisted of (+3)17:0/15:0-PG (*red*) and lesser amounts of 17:0/(+3)15:0-PG (*blue*). *C*, the (+3)34:0-PG peak consisted of (+3)19:0/15:0-PG (*red*) with a small percentage of 19:0/(+3)15:0-PG (*blue*). Panels *D*–*H* analyze the isotopic peaks from Figure 4*C*. *D*, the (+3)31:0-PG peak consisted of (+3)15:0/16:0-PG (*red*). *E*, the (+4)31:0-PG peak consisted of 17:0/(+4)14:0-PG (*blue*) and (+4)16:0/15:0-PG (*red*) combinations. The *black peaks* are contamination from the +1 natural abundance ^13^C isotope peak arising from the adjacent and more abundant (+3)31:0-PG peak (Fig. 4*C*). *F*, the (+7)31:0-PG peak consisted of (+3)17:0/(+4)14:0-PG (*blue*) and (+4)16:0/(+3)15:0-PG (*red*). *G*, the (+3)32:0-PG peak consisted of 17:0/(+3)15:0-PG (*blue*) and (+3)17:0/15:0-PG (*red*). *H*, the (+6)32:0-PG peak consisted of (+3)17:0/(+3)15:0-PG (*red*). PG, phosphatidylglycerol.

Each of the even number molecular species in Figure 4*B* were fragmented to determine the fatty acid composition and positional distribution. Acyl chains located at the 2-position fatty acid of the PG backbone are recovered at about twice the intensity as the 1-position fatty acid (30, 31, 32). The +3 peaks in 30:0-PG have an unlabeled 15:0 in the 2-position and *iso*-15:0 in the 1-position fatty acids (Fig. 5*A*). The 32:0-PG and 34:0-PG predominately have an *iso*-fatty acid in the 1-position paired with unlabeled 15:0 in the 2-position, although molecular species with an *iso*-15:0 in the 2-position paired with an unlabeled odd-chain fatty acid are also present (Fig. 5, *B* and *C*). The new odd number PG molecular species arising in cells grown without Ile (Fig. 4*C*) were fragmented to determine the acyl chain composition of these new peaks. The (+3) 31:0-PG consisted of unlabeled 16:0 in the 1-position paired with (+3) 15:0 (Fig. 5*D*). The (+4)31:0-PG was a mixture of Val-derived (+4)*i*16:0 paired with 15:0 or 17:0 paired with (+4)*i*14:0 (Fig. 5*E*). The (+7)31:0-PG was a mixture of (+3)*i*17:0 paired with (+4)*i*14:0 and (+4)*i*16:0 paired with (+3)*i*15:0 (Fig. 5*F*). The (+3)32:0-PG peak has one *iso* fatty acid (Fig. 5*G*), and the (+6)32:0-PG peak has two *iso* fatty acids (Fig. 5*H*). Thus, the absence of Ile in the medium led to a large increase in the incorporation of Leu-derived *iso*-odd-chain fatty acids in the PG molecular species. Val incorporation increased in the 31:0-PG molecular species in the absence of Ile but remained a quantitatively less important fatty acid precursor and was not elongated past *i*16:0.

### Acyl-CoA pool composition controls fatty acid structure

**Figure 6:**
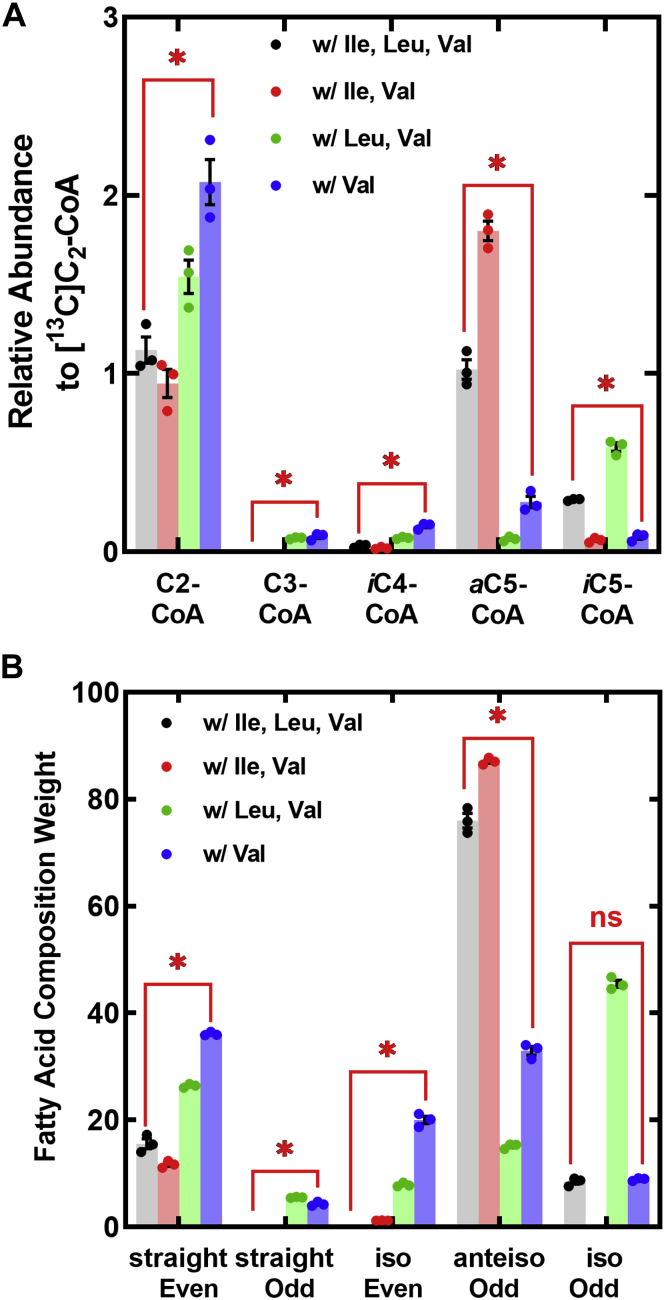
**Alteration in acyl-CoA and fatty acid composition in media lacking branched-chain amino acids.** Strain AH1263 was grown in defined media, and samples were taken at midlog phase. *A*, quantification of the CoA pool composition by LC–MS/MS using [^13^C]C_2_-CoA as an internal standard. *B*, the weight percent of each fatty acid produced was determined by gas LC of the derived methyl esters quantified using a flame ionization detector. Triplicate biological replicates were analyzed. A two-tailed Student's *t* test was used to compare the two indicated groups. ∗*p* < 0.005.

The impact of each of the four extracellular amino acid supplementation conditions on the abundance of acyl-CoA primers was analyzed by LC–MS/MS using three biological replicates (Fig. 6*A*). The removal of Leu from the medium reduces *i*C_5_-CoA and changes the *a*C_5_-CoA:*i*C_5_-CoA ratio from 3.5 to 27.3. The removal of Ile causes a marked reduction in *a*C_5_-CoA coupled with a large increase in *i*C_5_-CoA leading to a *a*C_5_-CoA:*i*C_5_-CoA ratio of 0.012. In the absence of both Ile and Leu, the overall concentration of branched-chain precursors is lower, but the *a*C_5_-CoA:*i*C_5_-CoA ratio of 3.4 is the same as in defined media. This result shows that the Ile is preferentially metabolized by the Bkd pathway in the absence of extracellular amino acids ruling out amino acid transporters as the selectivity filter. C_3_-CoA is not detected in defined media, but it contributes to the CoA thioester pool in media lacking Ile. The removal of Ile also results in an increase in the intracellular concentration of C_2_-CoA (Fig. 6*A*).

Gas chromatography reveals the close correlation between the acyl-CoA primer pool and fatty acid compositions (Fig. 6*B* and Table S1). The fatty acid composition is dominated by *anteiso* fatty acid in defined medium, and Leu removal causes an increase in *anteiso*-odd fatty acids and a decrease in *iso*-odd acyl chains. The removal of Ile from the medium resulted in a large increase in *iso* fatty acids and a reduction in *anteiso* acyl chains. Removal of both Leu and Ile from the medium returned the *anteiso:iso* ratio in branched-chain fatty acids to that of media containing Ile and Leu, but the overall abundance of odd-chain fatty acids is lower. There is also an increase in *iso*-even-chain fatty acids (*iso*-14:0 and *iso*-16:0) derived from Val under conditions where extracellular Ile is absent (Fig. 6*B* and Table S1). Also, odd straight-chain fatty acids are found in cells grown without Ile (Table S1) that arise from FabH utilization of the propionyl-CoA pool that is detected in the absence of Ile. However, the largest increases arising from Ile removal are in even-numbered fatty acids that are not derived from amino acid metabolism (Fig. 6*B*). This correlates with a change in the C_2_-CoA:C_5_-CoA ratio from 0.86 in defined medium to 5.7 in the absence of extracellular Ile and Leu (Fig. 6*A*). These straight-chain fatty acids are primarily 18:0 and 20:0 (Table S1), leading to an increase in 33:0-PG and 35:0-PG and an increase in the average carbon number of the PG molecular species. The close link between the acyl-CoA abundance and the fatty acid compositions is evident comparing Figure 6, *A* and *B*. In the absence of extracellular Ile, *S. aureus* compensates by switching to the synthesis of fatty acids derived from C_2_-CoA that are incorporated into the 1-position of PG. These data suggest that the composition of the acyl-CoA primer pool is the major determinant of fatty acid composition and not the selectivity of FabH.

### Impact of amino acids on the acyl-ACP pool

**Figure 7:**
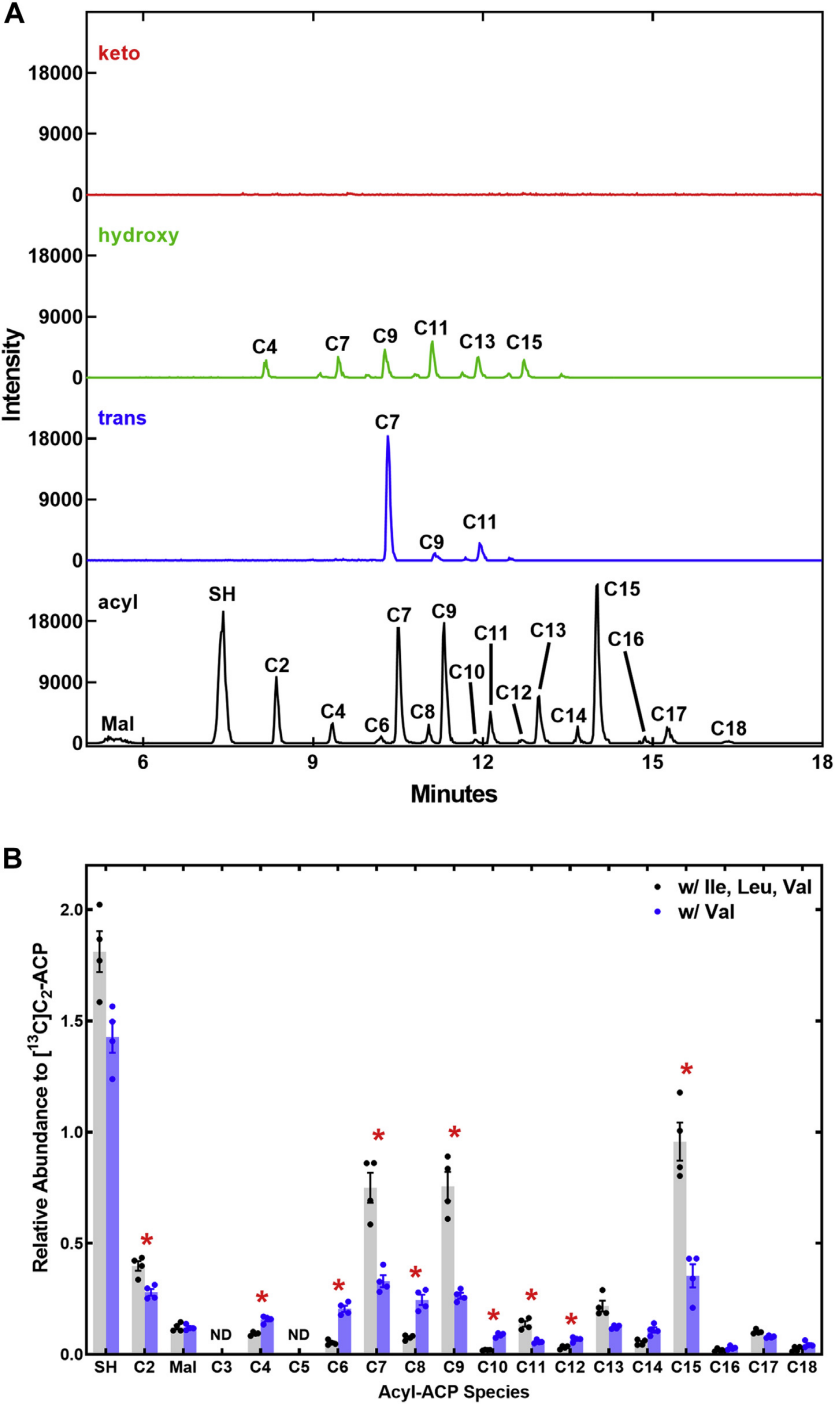
**Acyl-ACP pool composition in *Staphylococcus aureus* in the presence and absence of Ile/Leu.***A*, representative LC-MS/MS traces showing the profiles of 3-ketoacyl-ACP, 3-hydroxyacyl-ACP, *trans*-2-enoyl-ACP and acyl-ACP pools in *S. aureus* grown in defined media. The peaks are labeled with the carbon number of the intermediate acyl chain. *B*, quantification of the acyl-ACP pool composition in *S. aureus* grown in defined medium compared to cells grown in medium without Ile and Leu. A two-tailed Student's *t* test was used to compare the two indicated groups. ∗*p* < 0.005. ACP, acyl carrier protein.

The removal of Ile from the medium triggers the synthesis of even-chain fatty acids not derived from branched-chain amino acids. We analyzed the levels of FASII intermediates in *S. aureus* to determine the rate-limiting steps in FASII and Ile-dependent alteration in the levels of pathway intermediates. We used a proteomics workflow adapted from Nam *et al.* (38) to prepare acyl-ACP fragments and performed four separate LC–MS/MS experiments on each biological replicate to profile all potential 3-ketoacyl-ACP, *trans*-2-enoyl-ACP (*t*C_x_-ACP), 3-hydroxyacyl-ACP (*h*C_x_-ACP), and acyl-ACP (C_x_-ACP) intermediates. [^13^C_2_]Acetyl-ACP was spiked into the samples at the beginning of the workflow and used as the internal standard to normalize the levels of intermediates in each biological replicate. The raw ion current data from representative analytic experiments to determine the acyl-ACP levels in *S. aureus* grown in defined medium is shown to illustrate the primary data (Fig. 7*A*). We did not detect a pool of ketoacyl-ACP. Control experiments using FabH to generate 3-ketoC_4_-ACP show that this intermediate is recovered by our workflow suggesting that these intermediates are rapidly metabolized by FabG *in vivo*. There was a series of odd-chain *h*C_7+2n_-ACP detected (Fig. 7*A*). The relative abundance of these *h*C_7+2n_-ACP intermediates was 10-fold lower than the abundance of the corresponding acyl-ACPs. *t*C_7_-ACP was the major *trans* intermediate detected with levels of other *trans* intermediates being low or below the level of detection (Fig. 7*A*). The relative abundance of *t*C_7_-ACP in the pathway suggests that metabolism of the first *trans* intermediate is the slowest step catalyzed by FabI in the pathway. Odd-carbon acyl-ACPs are the major pathway intermediates detected (Fig. 7*A*) illustrating the preferential use of branched-chain FabH substrates when Ile and Leu are present. Nonesterified ACP (ACPSH) is a major pool component with malonyl-ACP and acetyl-ACP also detected. There are also low signals for even-chain acyl-ACP detected in cells grown in defined medium (Fig. 7*A*). The detection of these even-chain acyl-ACPs in the pathway is consistent with the essential function of FASII to supply C_8_-ACP for lipoic acid biosynthesis (39, 40, 41) coupled with the low levels of these fatty acids in the PG molecular species grown in defined medium. C_7_-ACP is the first odd intermediate and arises from the FabH condensation of a C_5_-CoA with malonyl-ACP. C_15_-ACP was the next most abundant acyl-ACP, and it is the primary PlsC substrate. The high relative abundance of acyl-ACP intermediates suggests that FabF is the slow step in each round of elongation in *S. aureus* FASII.

### Ile control of the acyl-ACP pool

The major shift in membrane fatty acid composition arising from the absence of Ile from the growth medium is the sharp increase in straight-chain even-carbon fatty acids. We compared the acyl-ACP pool composition of *S. aureus* grown in defined medium to growth in media lacking Ile and Leu. Even-carbon straight-chain acyl-ACP intermediates rose significantly in samples obtained from cells grown without Ile and Leu. This increase was coupled with a reduction in odd-chain acyl-ACP (Fig. 7*B*). These changes in pathway intermediate abundance reflect the alteration in fatty acid composition under these growth conditions. *S. aureus* FabH utilizes C_2_-CoA as a substrate (27, 28, 42), so FabH initiation with C_2_-CoA is the most likely origin of the elevated C_4_-ACP pool found in the absence of Ile and Leu. However, we cannot rule out an alternate pathway *via* acetyl-ACP condensation with malonyl-ACP by FabF, although how acetyl-ACP would be formed in *S. aureus* is unknown. The acyl-CoA:ACP transacylase activity of FabH may be responsible for acetyl-ACP, but there is no evidence for C_5_-ACP in the profile. C_5_-CoA is the preferred FabH substrate; therefore, C_5_-ACP should be present if there was significant FabH transacylation *in vivo*.

## Discussion

**Figure 8:**
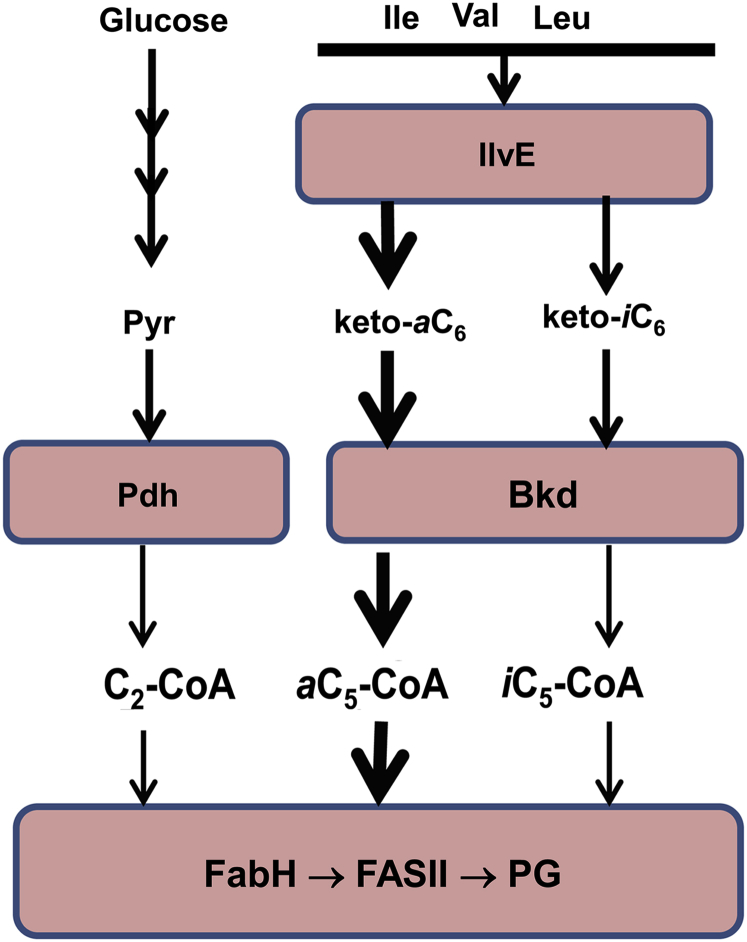
**Pathways responsible for the generation of short-chain acyl-CoA precursors.** Pyruvate (Pyr) arising from glycolysis is converted to C_2_-CoA by pyruvate dehydrogenase (Pdh) and is the precursor for even-numbered straight-chain fatty acids. There are other sources of C_2_-CoA including C_2_-CoA synthetase and phosphotransacetylase that are not shown. Extracellular branched-chain amino acids are transaminated by IlvE giving rise to the respective ketoacids. Alternately, the ketoacids are generated by the *de novo* branched-chain amino acid biosynthetic pathway. These ketoacids are decarboxylated to their respective acyl-CoAs by branched-chain ketoacid dehydrogenase (Bkd) to form 2-methyl-butyryl-CoA (*a*C_5_-CoA) and isobutyryl-CoA (*i*C_5_-CoA). Valine is a poor substrate for the Bkd pathway, whereas Ile is the major amino acid metabolized. The ratio of short-chain acyl-CoA primers available to FabH is a major determinant of fatty acid composition. The *anteiso* branched-chain odd-carbon fatty acids arise from *a*C_5_-CoA, *iso* branched-chain odd-carbon fatty acids are from *i*C_5_-CoA, straight-chain even-carbon fatty acids come from C_2_-CoA, and the trace of *iso* branched-chain even-carbon fatty acids are derived from Val.

Our results reveal the key role of branched-chain amino acid metabolism in determining the structure of membrane phospholipids in *S. aureus* (Fig. 8). Extracellular Ile and Leu have a major impact on the supply of FabH precursors to initiate fatty acid synthesis. The C_5_-CoA pool arises from the transamination of Ile and Leu followed by the formation of *a*C_5_-CoA and *i*C_5_-CoA by Bkd (Fig. 8). The ratio of *a*C_5_-CoA:*i*C_5_-CoA is 3.5 at equal extracellular Ile and Leu concentrations illustrating that Ile is the preferred substrate for the Bkd pathway. The Bkd selectivity for Ile is not stringent because removal of Ile from the media results in a large increase in *i*C_5_-CoA and *iso*-odd-chain fatty acids in membrane phospholipids. In contrast, Val is a poor substrate for the Bkd pathway and makes a minor contribution to fatty acid structure, even when it is the only extracellular branched-chain amino acid available (Fig. 8). The *a*C_5_-CoA:*i*C_5_-CoA ratio is 3.4 in the absence of extracellular Ile/Leu, meaning that the Bkd pathway has the same selectivity for substrates generated by *de novo* biosynthesis. C_2_-CoA is a major component of the acyl-CoA pool and arises from pyruvate dehydrogenase. Activation of acetate by C_2_-CoA synthetase and acetate kinase also supports the C_2_-CoA pool. Our metabolomics measurements show that the absence of branched-chain amino acids in the medium leads to a 75% decrease in the abundance of C_5_-CoA coupled with an increase in the amount of C_2_-CoA. This increases the C_2_:C_5_-CoA ratio from 0.86 in the presence of branched-chain amino acids to 5.7 in their absence leading to enhanced initiation of FASII using the C_2_-CoA primer. However, the selectivity of *S. aureus* FabH for C_5_-CoA (27, 28, 42) ensures there is always branched-chain acyl-ACP in the metabolic pipeline for the synthesis of either *a*15:0-ACP or *i*15:0-ACP for PlsC to place into the 2-position of the glycerol backbone. Although *i*14:0 derived from Val is used for the 2-position when Ile and Leu are absent from the medium, 15:0 carbon branched acyl chains dominate the 2-position of the phospholipid molecular species under all growth conditions studied. Our metabolomic datasets are consistent with the substrate selectivity exhibited by *S. aureus* FabH for C_2_-CoA and C_5_-CoA *in vitro* (27, 28, 42). In summary, our results point to the CoA thioester primer pool composition as being the primary determinant of the fatty acid composition of *S. aureus*. This means that changes in membrane phospholipid structure in *S. aureus* triggered by environmental conditions or signaling pathways are driven by changes in branched-chain amino acid metabolism that in turn impacts the acyl-CoA FabH primer pool.

Branched-chain amino acids are at the intersection of metabolism and virulence in *S. aureus* (43, 44, 45, 46, 47). Their levels (primarily Ile) are sensed by the CodY transcriptional regulator that controls a suite of genes involved in amino acid biosynthesis and virulence. When environmental Ile is limiting, the direct repression of alpha-toxin, hyaluronidase, and Panton–Valentine leucocidin by CodY is relaxed (48), and Δ*codY* strains are hypervirulent (49). *S. aureus* encounters limited quantities of branched-chain amino acids at the infection site, where they compete with the host for these essential nutrients (43). Consistent with this view, *S. aureus* phospholipid structure at the infection site is mimicked in the laboratory by a low Ile environment (36, 37). Similar effects on fatty acid composition are noted in *S. aureus* strains lacking branched-chain amino acid transporters (15) that reduce the availability of Ile for FASII and impacts virulence (50). Exogenous straight-chain fatty acids are incorporated into the 1-position of *S. aureus* phospholipids and suppress straight-chain synthesis (24, 33). This effect also arises when Ile is limiting and likely driven by changes in the supply of FabH primers. In future work studying the adaption of membrane composition to changes in the environment, measurement of the FabH precursor pool composition will be a requisite experiment to determine the impact of altered branched-chain amino acid metabolism. Whereas the ratio of straight:*anteiso*:*iso* fatty acids is governed by the composition of the acyl-CoA primer pool, chain length control remains a property governed by FASII components and their interaction with acyltransferases (Fig. 1).

## Experimental procedures

### Strains, materials, and media

*S. aureus* strain AH1263 was a USA300-014 genotype. [^13^C_6_]Isoleucine, [*d*_3_]leucine, and] [^13^C_5_,^15^N]valine were from Cambridge Isotope Laboratories, Inc. [^13^C_5_,^15^N]valine, rather than [^13^C_5_]valine, was used because it was less expensive, and the presence of ^15^N has no impact on the experiment. [^13^C_2_]C_2_-CoA was from Millipore Sigma. Defined media components and other reagents were purchased from Millipore Sigma or Thermo Fisher Scientific. All MS reagents are of HPLC grade or better. The defined media used in this work consist of M9 salts supplemented with amino acids and other nutrients as outlined in Table S2. The growth characteristics of *S. aureus* strain AH1263 in defined media lacking each of the branched-chain amino acids are illustrated in Figure S4. Like other *S. aureus* strains, wildtype strain AH1263 requires extracellular Val as a supplement in defined media (47, 51). Growth when Leu was removed was slower than in the other conditions as reported previously, but removal of either Ile or Ile + Leu did not affect growth (47, 51) (Fig. S4). The Leu growth phenotype arises from the Ile repression of branched-chain amino acid synthesis by CodY (47).

### Branched-chain amino acid growth experiments

An overnight culture of strain AH1263 was grown in defined medium at 37 °C (Table S2). The cells were collected by centrifugation at 4000*g* for 10 min at room temperature, washed with defined medium lacking Ile, Leu, and Val, and again collected by centrifugation. The cell pellets were resuspended in defined medium lacking Ile, Leu, and Val. The cell suspensions were used to inoculate strain AH1263 into the defined medium being used for the experiment to an absorbance of 0.05 at 600 nm. The growth rates of strain AH1263 in the presence or the absence of branched-chain amino acids are shown in Figure S4. In the metabolomics experiments, the cell cultures were grown to early log phase at an absorbance of 0.6 at 600 nm, at which point the cells were collected and extracted using the various workflows for the analysis of the different metabolomes.

Cells were labeled with heavy amino acids by collecting cells grown at 37 °C to an absorbance of 0.5 at 600 nm in defined medium by centrifugation at 4000*g* for 10 min at room temperature. The cell pellets were washed with defined medium lacking Ile, Leu, and Val and were collected by centrifugation. The cell pellets were resuspended in defined medium with the normal concentrations of Ile, Leu, and Val being replaced by [^13^C_6_]isoleucine, [*d*_3_]leucine, and [^13^C_5_,^15^N]valine. In the second labeling experiment, the cells were split in medium containing 50 μg/ml [*d*_3_]leucine and 20 μg/ml [^13^C_5_,^15^N]valine either with or without 50 μg/ml Ile. These cultures were incubated at 37 °C, and samples were taken at 10, 20, or 40 min for metabolomic analyses.

### Acyl-CoA MS

The cell pellet from 10 ml of culture was resuspended in 2 ml methanol and 1 ml water and incubated on ice for 30 min. Chloroform (1.5 ml) and water (1.2 ml) were added to remove the lipids. The aqueous top layer was collected, and 30 pmol of [^13^C_2_]C_2_-CoA (Millipore Sigma) was added, and the samples were dried overnight in a Savant Speedvac Concentrator SPD 1010 (Thermo Fisher Scientific). The dried sample was resuspended in 300 μl water, and 20 μl aliquots were used for analysis. Acyl-CoAs were analyzed using a Shimadzu Prominence UFLC attached to a QTrap 4500 equipped with a Turbo V ion source (Sciex). Samples were injected onto an Acquity UPLC HSS C18, 2.5 μm, 3.0 × 150 mm column at 40 °C (Waters) using a flow rate of 0.2 ml/min. Solvent A was 10 mM ammonium acetate, pH 6.8, and solvent B was 95% acetonitrile + 10 mM ammonium acetate, pH 6.8. The HPLC program was the following: starting solvent mixture of 83% A/17% B, 0 to 2 min isocratic with 17% B; 2 to 15 min linear gradient to 50% B; 15 to 20 min linear gradient to 95% B; 20 to 29 min isocratic with 95% B; 29 to 30 min linear gradient to 17% B; and 30 to 35 min isocratic with 17% B. The QTrap 4500 was operated in the positive mode, and the ion source parameters were ion spray voltage, 5500 V; curtain gas, 15 psi; temperature, 400 °C; collision gas, medium; ion source gas 1, 15 psi; ion source gas 2, 20 psi; declustering potential, 60 V; and collision energy, 45 V. Multiple reaction monitoring (MRM) is a specific and sensitive technique using a triple quadrupole mass spectrometer to quantify compounds in complex mixtures. MRMs are reported as two masses. The Q1 mass is for the intact molecule, and the Q3 mass is for the daughter ion being monitored. The Q1/Q3 masses are CoA, 768.1/261.1; C_2_-CoA, 810.1/303.1; C_3_-CoA, 824.1/317.1; *i*C_4_-CoA and C_4_-CoA, 838.1/331.1; *i*C_5_-CoA and *a*C_5_-CoA, 852.1/345.1; malonyl-CoA, 854.1/347.1; (+5)*a*C_5_-CoA, 857.1/350.1; (+3)*i*C_5_-CoA, 855.1/348.1; (+4)*i*C_4_-CoA, 842.1/335.1; and [^13^C_2_]C_2_-CoA, 812.1/305.1. [^13^C_2_]C_2_-CoA was used as the internal standard. The system was controlled by the Analyst software (Sciex) and analyzed with MultiQuant 3.0.2 software (Sciex). Peaks corresponding to individual acyl-CoA species were quantified relative to the internal standard.

### Lipid MS

Lipids were extracted from 5 ml of culture (52), and the dried lipids were suspended in chloroform/methanol (1:1). PG was analyzed using a Shimadzu Prominence UFLC attached to a QTrap 4500 equipped with a Turbo V ion source. Samples were injected onto an Acquity UPLC BEH HILIC, 1.7 μm, 2.1 × 150 mm column (Waters) at 45 °C with a flow rate of 0.2 ml/min. Solvent A was acetonitrile, and solvent B was 15 mM ammonium formate, pH 3.0. The HPLC program was the following: starting solvent mixture of 96% A/4% B; 0 to 2 min, isocratic with 4% B; 2 to 20 min, linear gradient to 80% B; 20 to 23 min, isocratic with 80% B; 23 to 25 min, linear gradient to 4% B; and 25 to 30 min, isocratic with 4% B. The QTrap 4500 was operated in the Q1 negative mode. The ion source parameters for Q1 were as follows: ion spray voltage, −4500 V; curtain gas, 25 psi; temperature, 350 °C; ion source gas 1, 40 psi; ion source gas 2, 60 psi; and declustering potential, −40 V. The system was controlled by the Analyst software. The sum of the areas under each peak in the mass spectra was calculated, and the percentage of each molecular species present was calculated with LipidView software (Sciex).

Samples were also introduced to the QTrap 4500 by direct injection to perform product scans to verify the fatty acids present in a particular PG molecular species. The ion source parameters for negative mode product scan were ion spray voltage, −4500 V; curtain gas, 10 psi; collision gas, medium; temperature, 270 °C; ion source gas 1, 10 psi; ion source gas 2, 15 psi; declustering potential, −40 V; and collision energy, −50 V.

### Acyl-ACP MS

Samples were prepared for acyl-ACP quantification as previously described (53) with modifications. One milliliter aliquots of cultures at midlog phase growth were removed to a 1.5 ml Eppendorf tube containing 250 μl ice cold 10% trichloroacetic acid. The tubes were inverted and kept on ice. Harvested cells were washed with acetone, dried, and then stored at −80 °C. Pellets were resuspended in 100 μl of lysis buffer (50 mM sodium phosphate buffer, pH 7.2, 1 mM ascorbic acid, 2 mM EDTA, and 6 M urea). Lysis buffer was prepared just prior to use. [^13^C_2_]Acetyl-ACP was added as an internal standard (1 μl of 0.0125 μg/μl [^13^C_2_]acetyl-ACP). A chloroform/methanol extraction was performed by adding 400 μl methanol, followed by 100 μl of chloroform. Samples were sonicated in a sonication bath (Fisher Scientific CPXH Series 1.9L) for 10 min at room temperature to resuspend. Phases were separated by adding 300 μl of 0.2 mM formate buffer, pH 3.9. The upper phase was discarded, and 300 μl of methanol was added to the remaining sample. The precipitated proteins were collected by centrifugation for 5 min at top speed in an Eppendorf microfuge (20,000*g*), and the pellets were washed with 300 μl of methanol and dried. The dried pellets were resuspended in 10 μl of 100 mM sodium phosphate buffer, pH 6.5, and sonicated for 10 min at room temperature in an ultrasonic bath. Insoluble debris was removed by centrifugation at top speed in a microfuge. A 5 μl aliquot of the supernatant was removed to a clean tube. Proteins were digested by adding 0.5 μg endoproteinase Asp-N (Millipore Sigma) in 10 μl of 100 mM Tris, pH 7.5 (38). Samples were incubated 1 h at room temperature. Proteolysis was quenched with 15 μl methanol.

Acyl-ACP species were analyzed using a Shimadzu Prominence UFLC attached to a QTrap 4500 equipped with a Turbo V ion source. Samples were injected onto an Acquity UPLC Discovery B10 Wide Pore C18, 3 μm, 2.1 × 100 mm column (Millipore Sigma) at 25 °C with a flow rate of 0.2 ml/min. Solvent A was 10 mM ammonium formate, pH 3.6, in 10% acetonitrile, and solvent B was 10 mM ammonium formate, pH 3.6, in 90% acetonitrile. The HPLC program was the following: starting solvent mixture of 100% A/0% B; 0 to 4 min, linear gradient to 10% B; 4 to 12 min, linear gradient to 100% B; 12 to 17 min, isocratic with 100% B; 17 to 20 min, linear gradient to 0% B; and 20 to 25 min, isocratic with 0% B. The QTrap 4500 was operated in the positive mode, and the ion source parameters were ion spray voltage, 5500 V; curtain gas, 30 psi; temperature, 400 °C; collision gas, medium; ion source gas 1, 25 psi; and ion source gas 2, 30 psi. The Q1/Q3 MRM parameters for [^13^C_2_]acetyl-ACP were 718.3/350.2 with a declustering potential of 80 V and a collision energy of 40 V. The analytic parameters for all the ACP pathway intermediates are listed in Tables S3–S6. The system was controlled by the Analyst software and analyzed with MultiQuant 3.0.2 software (Sciex). [^13^C_2_]C_2_-ACP was used as the internal standard. Peaks corresponding to individual acyl-ACP species were quantified relative to the internal standard.

### Fatty acid gas chromatography

Fatty acid methyl esters were prepared using methanol/hydrochloric acid. The fatty acid methyl esters were analyzed by a Hewlett–Packard model 5890 gas chromatograph equipped with a flame ionization detector and separated on 30 m × 0.536 mm × 0.50 μm DB-225 capillary column (Agilent). The injector was set at 250 °C, and the detector was at 300 °C. The temperature program was as follows: initial temperature of 70 °C for 2 min, rate of 20 °C/min for 5 min (final 170 °C), rate of 2 °C/min for 10 min (final 190 °C), hold at 190 °C for 5 min, rate of 2 °C/min for 15 min (final 220 °C), and hold at 220 °C for 5 min. The identity of fatty acid methyl esters was determined by comparing their retention times with fatty acid methyl ester standards (Matreya). The compositions were expressed as weight percentages.

### Preparation of [^13^C_2_]acetyl-ACP

^13^C-labeled acetyl-ACP was synthesized by the previously described method for crotonyl-ACP synthesis (54) with slight modifications. His_6_-tagged *E. coli* apo-ACP was expressed using the pET-15b plasmid transformed into BL21(DE3) cells. After induction with 1 mM IPTG for 3 h at 37 °C, cells were lysed and apo-ACP was purified using nickel affinity chromatography (GoldBio). Pooled purified protein was dialyzed overnight in 20 mM Tris, pH 7.5, to remove the imidazole. The His_6_ tag was then cleaved by thrombin treatment (Millipore) and separated from the apo-ACP by nickel chromatography. [^13^C_2_]Acetyl-ACP was synthesized by incubating 300 μM purified apo-ACP with 2 μM *E. coli* ACP synthase and 600 μM [^13^C_2_]acetyl CoA (Millipore Sigma) in 50 mM Tris, pH 7, with 10 mM MgCl_2_ for 2 h at 37 °C. The His_6_-tagged [ACP]synthase was subsequently removed from the reaction by nickel affinity chromatography. The MgCl_2_ and excess CoA were removed using a PD-10 desalting column. The [^13^C_2_]acetyl-ACP was eluted in 20 mM Bis–Tris, pH 6, with 200 mM NaCl and concentrated using a 3 kDa cutoff Amicon Ultra centrifugal filter. The concentrated standard was quantified using the Bicinchoninic Acid Protein Assay Kit (Pierce).

### Statistical analysis

Measurements are means ± SEM of biological replicates. The Student's *t* test was used to compare two groups using GraphPad Prism software (version 9.1.2; GraphPad Software, Inc).

## Data availability

All study data are included in the article.

## Supporting information

This article contains supporting information.

## Conflict of interest

The authors declare they have no conflicts of interest with the contents of this article.

## References

[1] Parsons J.B., Rock C.O. (2013). Bacterial lipids: Metabolism and membrane homeostasis. Prog. Lipid Res..

[2] Zhang Y.-M., Rock C.O. (2008). Membrane lipid homeostasis in bacteria. Nat. Rev. Microbiol..

[3] de Mendoza D., Klages Ulrich A., Cronan J.E. (1983). Thermal regulation of membrane fluidity in *Escherichia coli*. Effects of overproduction of β-ketoacyl-acyl carrier protein synthase I. J. Biol. Chem..

[4] Aguilar P.S., Lopez P., de Mendoza D. (1999). Transcriptional control of the low-temperature-inducible *des* gene, encoding the Δ5 desaturase of *Bacillus subtilis*. J. Bacteriol..

[5] Ulrich A.K., de Mendoza D., Garwin J.L., Cronan J.E. (1983). Genetic and biochemical analyses of *Escherichia coli* mutants altered in the temperature-dependent regulation of membrane lipid composition. J. Bacteriol..

[6] de Mendoza D., Cronan J.E. (1983). Thermal regulation of membrane lipid fluidity in bacteria. Trends Biochem. Sci..

[7] Garwin J.L., Klages A.L., Cronan J.E. (1980). β-Ketoacyl-acyl carrier protein synthase II of *Escherichia coli*. Evidence for function in the thermal regulation of fatty acid synthesis. J. Biol. Chem..

[8] Babu M.M., Priya M.L., Selvan A.T., Madera M., Gough J., Aravind L., Sankaran K. (2006). A database of bacterial lipoproteins (DOLOP) with functional assignments to predicted lipoproteins. J. Bacteriol..

[9] Cybulski L.E., Martin M., Mansilla M.C., Fernandez A., de Mendoza D. (2010). Membrane thickness cue for cold sensing in a bacterium. Curr. Biol..

[10] Lu Y.J., Rock C.O. (2006). Transcriptional regulation of fatty acid biosynthesis in *Streptococcus pneumoniae*. Mol. Microbiol..

[11] Jerga A., Rock C.O. (2009). Acyl-acyl carrier protein regulates transcription of fatty acid biosynthetic genes via the FabT repressor in *Streptococcus pneumoniae*. J. Biol. Chem..

[12] Zhang Y.-M., Marrakchi H., Rock C.O. (2002). The FabR (YijC) transcription factor regulates unsaturated fatty acid biosynthesis in *Escherichia coli*. J. Biol. Chem..

[13] Saunders L.P., Sen S., Wilkinson B.J., Gatto C. (2016). Insights into the mechanism of homeoviscous adaptation to low temperature in branched-chain fatty acid-containing bacteria through modeling FabH kinetics from the foodborne pathogen *Listeria monocytogenes*. Front. Microbiol..

[14] Sen S., Sirobhushanam S., Johnson S.R., Song Y., Tefft R., Gatto C., Wilkinson B.J. (2016). Growth-environment dependent modulation of *Staphylococcus aureus* branched-chain to straight-chain fatty acid ratio and incorporation of unsaturated fatty acids. PLoS One.

[15] Kaiser J.C., Sen S., Sinha A., Wilkinson B.J., Heinrichs D.E. (2016). The role of two branched-chain amino acid transporters in *Staphylococcus aureus* growth, membrane fatty acid composition and virulence. Mol. Microbiol..

[16] Zhu K., Ding X., Julotok M., Wilkinson B.J. (2005). Exogenous isoleucine and fatty acid shortening ensure the high content of anteiso-C15:0 fatty acid required for low-temperature growth of *Listeria monocytogenes*. Appl. Environ. Microbiol..

[17] Giotis E.S., McDowell D.A., Blair I.S., Wilkinson B.J. (2007). Role of branched-chain fatty acids in pH stress tolerance in *Listeria monocytogenes*. Appl. Environ. Microbiol..

[18] Poger D., Caron B., Mark A.E. (2014). Effect of methyl-branched fatty acids on the structure of lipid bilayers. J. Phys. Chem. B.

[19] Mostofian B., Zhuang T., Cheng X., Nickels J.D. (2019). Branched-chain fatty acid content modulates structure, fluidity, and phase in model microbial cell membranes. J. Phys. Chem. B.

[20] Annous B.A., Becker L.A., Bayles D.O., Labeda D.P., Wilkinson B.J. (1997). Critical role of *anteiso*-C15:0 fatty acid in the growth of *Listeria monocytogenes* at low temperatures. Appl. Environ. Microbiol..

[21] Bajerski F., Wagner D., Mangelsdorf K. (2017). Cell membrane fatty acid composition of *Chryseobacterium frigidisoli* PB4(T), isolated from Antarctic glacier Forefield soils, in response to changing temperature and pH conditions. Front. Microbiol..

[22] Mansilla M.C., de Mendoza D. (2005). The *Bacillus subtilis* desaturase: A model to understand phospholipid modification and temperature sensing. Arch. Microbiol..

[23] Zhu K., Choi K.-H., Schweizer H.P., Rock C.O., Zhang Y.-M. (2006). Two aerobic pathways for the formation of unsaturated fatty acids in *Pseudomonas aeruginosa*. Mol. Microbiol..

[24] Parsons J.B., Frank M.W., Subramanian C., Saenkham P., Rock C.O. (2011). Metabolic basis for the differential susceptibility of Gram-positive pathogens to fatty acid synthesis inhibitors. Proc. Natl. Acad. Sci. U. S. A..

[25] Klein W., Weber M.H., Marahiel M.A. (1999). Cold shock response of *Bacillus subtilis*: Isoleucine-dependent switch in the fatty acid branching pattern for membrane adaptation to low temperatures. J. Bacteriol..

[26] Singh V.K., Hattangady D.S., Giotis E.S., Singh A.K., Chamberlain N.R., Stuart M.K., Wilkinson B.J. (2008). Insertional inactivation of branched-chain α-keto acid dehydrogenase in *Staphylococcus aureus* leads to decreased branched-chain membrane fatty acid content and increased susceptibility to certain stresses. Appl. Environ. Microbiol..

[27] Choi K.-H., Heath R.J., Rock C.O. (2000). β-Ketoacyl-acyl carrier protein synthase III (FabH) is a determining factor in branched-chain fatty acid biosynthesis. J. Bacteriol..

[28] Singh A.K., Zhang Y.-M., Zhu K., Subramanian C., Li Z., Jayaswal R.K., Gatto C., Rock C.O., Wilkinson B.J. (2009). FabH selectivity for *anteiso* branched-chain fatty acid precursors in low temperature adaptation in *Listeria monocytogenes*. FEMS Microbiol. Lett..

[29] Kreiswirth B.N., Lofdahl S., Betley M.J., O'Reilly M., Schlievert P.M., Bergdoll M.S., Novick R.P. (1983). The toxic shock syndrome exotoxin structural gene is not detectably transmitted by a prophage. Nature.

[30] Han X., Gross R.W. (1995). Structural determination of picomole amounts of phospholipids via electrospray ionization tandem mass spectrometry. J. Am. Soc. Mass Spectrom..

[31] Hsu F.F., Turk J. (2001). Studies on phosphatidylglycerol with triple quadrupole tandem mass spectrometry with electrospray ionization: Fragmentation processes and structural characterization. J. Am. Soc. Mass Spectrom..

[32] Mazzella N., Molinet J., Syakti A.D., Dodi A., Doumenq P., Artaud J., Bertrand J.C. (2004). Bacterial phospholipid molecular species analysis by ion-pair reversed-phase HPLC/ESI/MS. J. Lipid Res..

[33] Parsons J.B., Frank M.W., Jackson P., Subramanian C., Rock C.O. (2014). Incorporation of extracellular fatty acids by a fatty acid kinase-dependent pathway in *Staphylococcus aureus*. Mol. Microbiol..

[34] Robertson R.M., Yao J., Gajewski S., Kumar G., Martin E.W., Rock C.O., White S.W. (2017). A two-helix motif positions the lysophosphatidic acid acyltransferase active site for catalysis within the membrane bilayer. Nat. Struct. Mol. Biol..

[35] Tiwari K.B., Gatto C., Wilkinson B.J. (2018). Interrelationships between fatty acid composition, staphyloxanthin content, fluidity, and carbon flow in the *Staphylococcus aureus* membrane. Molecules.

[36] Frank M.W., Yao J., Batte J.L., Gullett J.M., Subramanian C., Rosch J.W., Rock C.O. (2020). Host fatty acid utilization by *Staphylococcus aureus* at the infection site. mBio.

[37] Teoh W.P., Chen X., Laczkovich I., Alonzo F. (2021). *Staphylococcus aureus* adapts to the host nutritional landscape to overcome tissue-specific branched-chain fatty acid requirement. Proc. Natl. Acad. Sci. U. S. A..

[38] Nam J.W., Jenkins L.M., Li J., Evans B.S., Jaworski J.G., Allen D.K. (2020). A general method for quantification and discovery of acyl groups attached to acyl carrier proteins in fatty acid metabolism using LC-MS/MS. Plant Cell.

[39] Christensen Q.H., Cronan J.E. (2010). Lipoic acid synthesis: A new family of octanoyltransferases generally annotated as lipoate protein ligases. Biochemistry.

[40] Zorzoli A., Grayczyk J.P., Alonzo F. (2016). *Staphylococcus aureus* tissue infection during sepsis is supported by differential use of bacterial or host-derived lipoic acid. PLoS Pathog..

[41] Teoh W.P., Resko Z.J., Flury S., Alonzo F. (2019). Dynamic relay of protein-bound lipoic acid in *Staphylococcus aureus*. J. Bacteriol..

[42] Qiu X., Choudhry A.E., Janson C.A., Grooms M., Daines R.A., Lonsdale J.T., Khandekar S.S. (2005). Crystal structure and substrate specificity of the β-ketoacyl-acyl carrier protein synthase III (FabH) from *Staphylococcus aureus*. Prot. Sci..

[43] Kaiser J.C., Heinrichs D.E. (2018). Branching out: Alterations in bacterial physiology and virulence due to branched-chain amino acid deprivation. mBio.

[44] Pohl K., Francois P., Stenz L., Schlink F., Geiger T., Herbert S., Goerke C., Schrenzel J., Wolz C. (2009). CodY in *Staphylococcus aureus*: A regulatory link between metabolism and virulence gene expression. J. Bacteriol..

[45] Brinsmade S.R. (2017). CodY, a master integrator of metabolism and virulence in Gram-positive bacteria. Curr. Genet..

[46] Waters N.R., Samuels D.J., Behera R.K., Livny J., Rhee K.Y., Sadykov M.R., Brinsmade S.R. (2016). A spectrum of CodY activities drives metabolic reorganization and virulence gene expression in Staphylococcus aureus. Mol. Microbiol..

[47] Kaiser J.C., King A.N., Grigg J.C., Sheldon J.R., Edgell D.R., Murphy M.E.P., Brinsmade S.R., Heinrichs D.E. (2018). Repression of branched-chain amino acid synthesis in *Staphylococcus aureus* is mediated by isoleucine via CodY, and by a leucine-rich attenuator peptide. PLoS Genet..

[48] Ibberson C.B., Jones C.L., Singh S., Wise M.C., Hart M.E., Zurawski D.V., Horswill A.R. (2014). *Staphylococcus aureus* hyaluronidase is a CodY-regulated virulence factor. Infect. Immun..

[49] Montgomery C.P., Boyle-Vavra S., Roux A., Ebine K., Sonenshein A.L., Daum R.S. (2012). CodY deletion enhances *in vivo* virulence of community-associated methicillin-resistant *Staphylococcus aureus* clone USA300. Infect. Immun..

[50] Kaiser J.C., Omer S., Sheldon J.R., Welch I., Heinrichs D.E. (2015). Role of BrnQ1 and BrnQ2 in branched-chain amino acid transport and virulence in *Staphylococcus aureus*. Infect. Immun..

[51] Lincoln R.A., Leigh J.A., Jones N.C. (1995). The amino acid requirements of *Staphylococcus aureus* isolated from cases of bovine mastitis. Vet. Microbiol..

[52] Bligh E.G., Dyer W.J. (1959). A rapid method of total lipid extraction and purification. Can. J. Biochem. Physiol..

[53] Noga M.J., Buke F., van den Broek N.J.F., Imholz N.C.E., Scherer N., Yang F., Bokinsky G. (2020). Posttranslational control of PlsB is sufficient to coordinate membrane synthesis with growth in *Escherichia coli*. mBio.

[54] Yao J., Maxwell J.B., Rock C.O. (2013). Resistance to AFN-1252 arises from missense mutations in *Staphylococcus aureus* enoyl-acyl carrier protein reductase (FabI). J. Biol. Chem..

