# Supplementary material for: Branched-chain amino acid metabolism controls membrane phospholipid structure in Staphylococcus aureus
Source: J Biol Chem. 2021 Sep 28;297(5):101255. doi: 10.1016/j.jbc.2021.101255 (PMC8524195; doi:10.1016/j.jbc.2021.101255)
Supplement: Tables S1–S6 and Figures S1–S4 [file mmc1.pdf]

Supplementary Materials

Branched-chain amino acid metabolism controls membrane phospholipid structure in *Staphylococcus aureus*

Matthew W. Frank, Sarah G. Whaley and Charles O. Rock

Tables S1 to S6
Figures S1 to S4

Table S1
Fatty acid composition of *S. aureus* grown with different branched-chain amino acids
Strain AH1263 was grown in either complete defined medium or in media lacking either Ile, Leu or Ile plus Leu as described under Experimental Procedures. Cells were extracted and the derived fatty acid methyl esters were resolved on a capillary gas chromatography column equipped with flame ionization detector for quantification. Triplicate biological samples were analyzed. Means ± S.E.M. were calculated using GraphPad/Prism software.

	w/ Ile, Leu, Val		w/ Ile, Val		w/ Leu, Val		w/ Val	
Fatty Acid	% Total	SEM	% Total	SEM	% Total	SEM	% Total	SEM
i14:0	ND*		ND		2.67	0.13	5.73	0.20
14:0	ND		ND		0.20	0.01	0.58	0.01
i15:0	3.45	0.30	ND		28.77	0.88	4.31	0.04
a15:0	39.04	0.98	40.28	0.78	11.67	0.29	24.51	1.01
i16:0	ND		0.61	0.01	3.12	0.05	7.59	0.50
16:0	2.46	0.20	2.04	0.33	2.80	0.08	2.51	0.04
i17:0	3.31	0.16	ND		10.91	0.54	1.30	0.07
a17:0	28.35	0.95	34.35	0.41	2.71	0.10	6.10	0.21
17:0	ND		ND		1.00	0.05	0.70	0.01
i18:0	ND		0.59	0.03	1.67	0.06	4.40	0.16
18:0	7.08	0.43	5.27	0.12	11.53	0.26	11.62	0.44
i19:0	1.70	0.02	ND		5.36	0.07	1.05	0.04
a19:0	8.58	0.19	12.47	0.36	0.74	0.05	2.35	0.06
19:0	ND		ND		4.53	0.09	3.59	0.21
i20:0	ND		ND		0.44	0.03	2.27	0.16
20:0	6.02	0.33	4.39	0.08	11.88	0.31	21.37	0.65

* ND means less than 0.2%.

Table S2
Composition of defined growth medium for *S. aureus*.

Components	Final Concentration
M9 Salts[a]	1X
$MgSO_4$	1 mM
$CaCl_2$	0.1 mM
Vitamin B1	0.0005%
Vitamin B3	32 μM
Glucose	0.4%
Biotin	0.1 μg/ml
Pantothenate	2 μg/ml
$FeCl_3$	10 μM
Citrate	6 μg/ml
$MnCl_2$	10 μg/ml
Lipoic Acid	0.1 μg/ml
Phenylalanine	15 μg/ml
Lysine-HCl	40 μg/ml
Arginine	200 μg/ml
Glycine	10 μg/ml
Serine	30 μg/ml
Proline	20 μg/ml
Hydroxyproline	20 μg/ml
Aspartic Acid	20 μg/ml
Threonine	20 μg/ml
Histidine	15 μg/ml
Cystine-2HCl	65 μg/ml
Tyrosine	24 μg/ml
Methionine	15 μg/ml
Asparagine	57 μg/ml
Glutamine	50 μg/ml
Valine	20 μg/ml
Leucine	50 μg/ml
Isoleucine	50 μg/ml

[a] M9 salts formula from Ref. (48).

Table S3
MRM table for the detection of acyl-ACP by mass spectrometry.

acyl-ACP	Q1[a]	Q3[b]	DP[c]	CE[d]
apo-ACP	334.2	203.1	60	15
holo-ACP	674.2	261.1	80	40
C2-ACP	716.3	303.2	85	40
Mal-ACP	760.2	347.2	85	45
C3-ACP	730.3	317.2	85	40
C4-ACP	744.3	331.2	85	40
C5-ACP	758.3	345.2	90	45
C6-ACP	772.3	359.2	90	45
C7-ACP	786.3	373.2	90	45
C8-ACP	800.4	387.3	90	45
C9-ACP	814.3	401.2	90	45
C10:1-ACP	826.4	413.3	90	45
C10-ACP	828.4	415.3	90	45
C11-ACP	842.3	429.2	90	45
C12:1-ACP	854.4	441.3	90	45
C12-ACP	856.4	443.3	90	45
C13-ACP	870.3	457.2	90	45
C14:1-ACP	882.4	469.3	90	45
C14-ACP	884.4	471.3	90	45
C15-ACP	898.3	485.2	90	45
C16:1-ACP	910.5	497.4	90	45
C16-ACP	912.5	499.4	90	45
C17-ACP	926.3	513.2	90	45
C18:1-ACP	938.5	525.4	90	45
C18-ACP	940.5	527.4	90	45
C19-ACP	954.3	541.2	90	45
C20:1-ACP	966.3	553.2	90	45
C20-ACP	968.3	555.2	90	45
C21-ACP	982.3	569.2	90	45

[a] Q1 is the parent *m/z*.
[b] Q3 is the product *m/z*.
[b] DP is declustering potential (V).
[c] CE is collision energy (V).

Table S4
MRM table for the detection of hydroxyacyl-ACP by mass spectrometry.

hydroxyacyl-ACP	Q1[a]	Q3[b]	DP[c]	CE[d]
*h*C4-ACP	760.3	347.2	85	40
*h*C5-ACP	774.3	361.2	90	45
*h*C6-ACP	788.3	375.2	90	45
*h*C7-ACP	802.3	389.2	90	45
*h*C8-ACP	816.3	403.2	90	45
*h*C9-ACP	830.3	417.2	90	45
*h*C10:1-ACP	842.4	429.3	90	45
*h*C10-ACP	844.4	431.3	90	45
*h*C11-ACP	858.4	445.3	90	45
*h*C12:1-ACP	870.4	457.3	90	45
*h*C12-ACP	872.4	459.3	90	45
*h*C13-ACP	886.4	473.3	90	45
*h*C14:1-ACP	898.4	485.3	90	45
*h*C14-ACP	900.4	487.3	90	45
*h*C15-ACP	914.4	501.3	90	45
*h*C16:1-ACP	926.5	513.4	90	45
*h*C16-ACP	928.5	515.4	90	45
*h*C17-ACP	942.5	529.4	90	45
*h*C18:1-ACP	954.5	541.4	90	45
*h*C18-ACP	956.5	543.4	90	45
*h*C19-ACP	970.5	557.4	90	45
*h*C20:1-ACP	982.5	569.4	90	45
*h*C20-ACP	984.5	571.4	90	45
*h*C21-ACP	998.5	585.4	90	45

[a] Q1 is the parent *m/z*.
[b] Q3 is the product *m/z*.
[b] DP is declustering potential (V).
[c] CE is collision energy (V).

Table S5
MRM table for the detection of *trans*-2-acyl-ACP by mass spectrometry.

trans-ACP	Q1[a]	Q3[b]	DP[c]	CE[d]
*t*C4-ACP	742.3	329.2	85	40
*t*C5-ACP	756.3	343.2	90	45
*t*C6-ACP	770.3	357.2	90	45
*t*C7-ACP	784.3	371.2	90	45
*t*C8-ACP	798.3	385.2	90	45
*t*C9-ACP	812.3	399.2	90	45
*t*C10:1-ACP	824.4	411.3	90	45
*t*C10-ACP	826.4	413.3	90	45
*t*C11-ACP	840.3	427.2	90	45
*t*C12:1-ACP	852.4	439.3	90	45
*t*C12-ACP	854.4	441.3	90	45
*t*C13-ACP	868.4	455.3	90	45
*t*C14:1-ACP	880.4	467.3	90	45
*t*C14-ACP	882.4	469.3	90	45
*t*C15-ACP	896.4	483.3	90	45
*t*C16:1-ACP	908.5	495.4	90	45
*t*C16-ACP	910.5	497.4	90	45
*t*C17-ACP	924.5	511.4	90	45
*t*C18:1-ACP	936.5	523.4	90	45
*t*C18-ACP	938.5	525.4	90	45
*t*C19-ACP	952.5	539.4	90	45
*t*C20:1-ACP	964.5	551.4	90	45
*t*C20-ACP	966.5	553.4	90	45
*t*C21-ACP	980.5	567.5	90	45

[a] Q1 is the parent *m/z*.
[b] Q3 is the product *m/z*.
[b] DP is declustering potential (V).
[c] CE is collision energy (V).

Table S6
MRM table for the detection of 3-ketoacyl-ACP by mass spectrometry.

3-ketoacyl-ACP	Q1[a]	Q3[b]	DP[c]	CE[d]
*k*C4-ACP	758.3	345.2	90	40
*k*C5-ACP	772.3	359.2	95	45
*k*C6-ACP	786.3	373.2	95	45
*k*C7-ACP	800.3	387.2	95	45
*k*C8-ACP	814.3	401.2	95	45
*k*C9-ACP	828.3	415.2	95	45
*k*C10:1-ACP	840.3	427.3	95	45
*k*C10-ACP	842.3	429.3	95	45
*k*C11-ACP	856.4	443.3	95	45
*k*C12:1-ACP	868.3	455.3	95	45
*k*C12-ACP	870.3	457.3	95	45
*k*C13-ACP	884.4	471.3	95	45
*k*C14:1-ACP	896.3	483.3	95	45
*k*C14-ACP	898.3	485.3	95	45
*k*C15-ACP	912.4	499.3	95	45
*k*C16:1-ACP	924.4	511.4	95	45
*k*C16-ACP	926.4	513.4	95	45
*k*C17-ACP	940.5	527.4	95	45
*k*C18:1-ACP	952.5	539.4	95	45
*k*C18-ACP	954.5	541.4	95	45
*k*C19-ACP	968.5	555.4	95	45
*k*C20:1-ACP	980.5	567.4	95	45
*k*C20-ACP	982.5	569.4	95	45
*k*C21-ACP	996.5	583.4	95	45

[a] Q1 is the parent *m/z*.
[b] Q3 is the product *m/z*.
[b] DP is declustering potential (V).
[c] CE is collision energy (V).

Phosphatidylglycerol (PG) Molecular Species

Carbon Number	*m/z*	Acyl Chain Composition	
		1-position	2-position
30:0	693.5	15:0	15:0
31:0	707.6	16:0	15:0
32:0	721.5	17:0	15:0
33:0	735.6	18:0	15:0
34:0	749.6	19:0	15:0
35:0	763.6	20:0	15:0

Structure of Phosphatidylglycerol (PG)

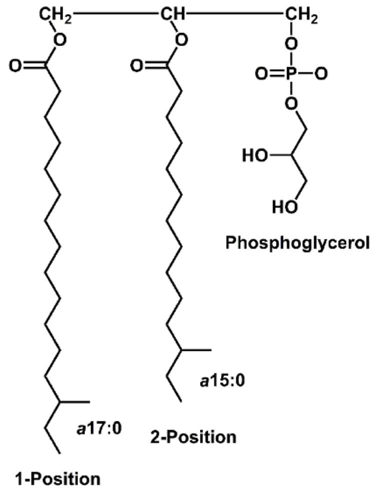

Figure S1. Structures of phosphatidylglycerol (PG) molecular species in *S. aureus* grown in defined medium. The table on the left lists the carbon number and *m/z* masses detected in the mass spectrometry experiments and the composition of the 1- and 2-position fatty acids in these molecular species. The chemical structure on the right shows the chemical structure of the major PG molecular species (32:0) with the 1- and 2-position indicated. In defined medium (with isoleucine present) the most abundant branched-chain fatty acids have *anteiso* branching.

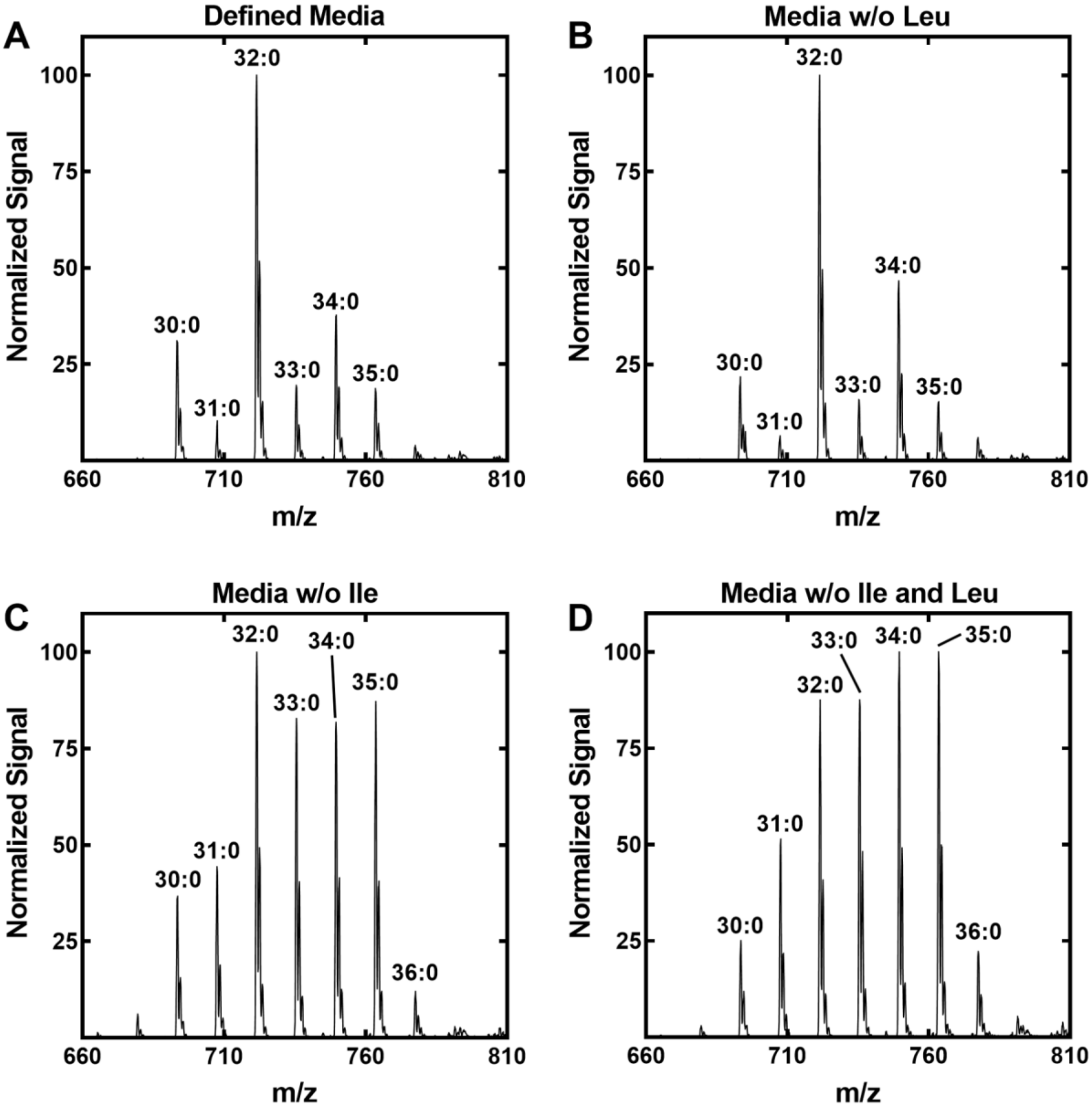

Figure S2. PG molecular species of *S. aureus* grown with different branched-chain amino acids. Strain AH1263 was grown in defined media as described in Experimental Procedures and representative mass spectra illustrating the PG molecular species composition are shown for each growth condition. *A*, complete defined medium containing Ile, Leu and Val. *B*, medium lacking Leu. *C*, medium lacking Ile. *D*, medium lacking Ile and Leu.

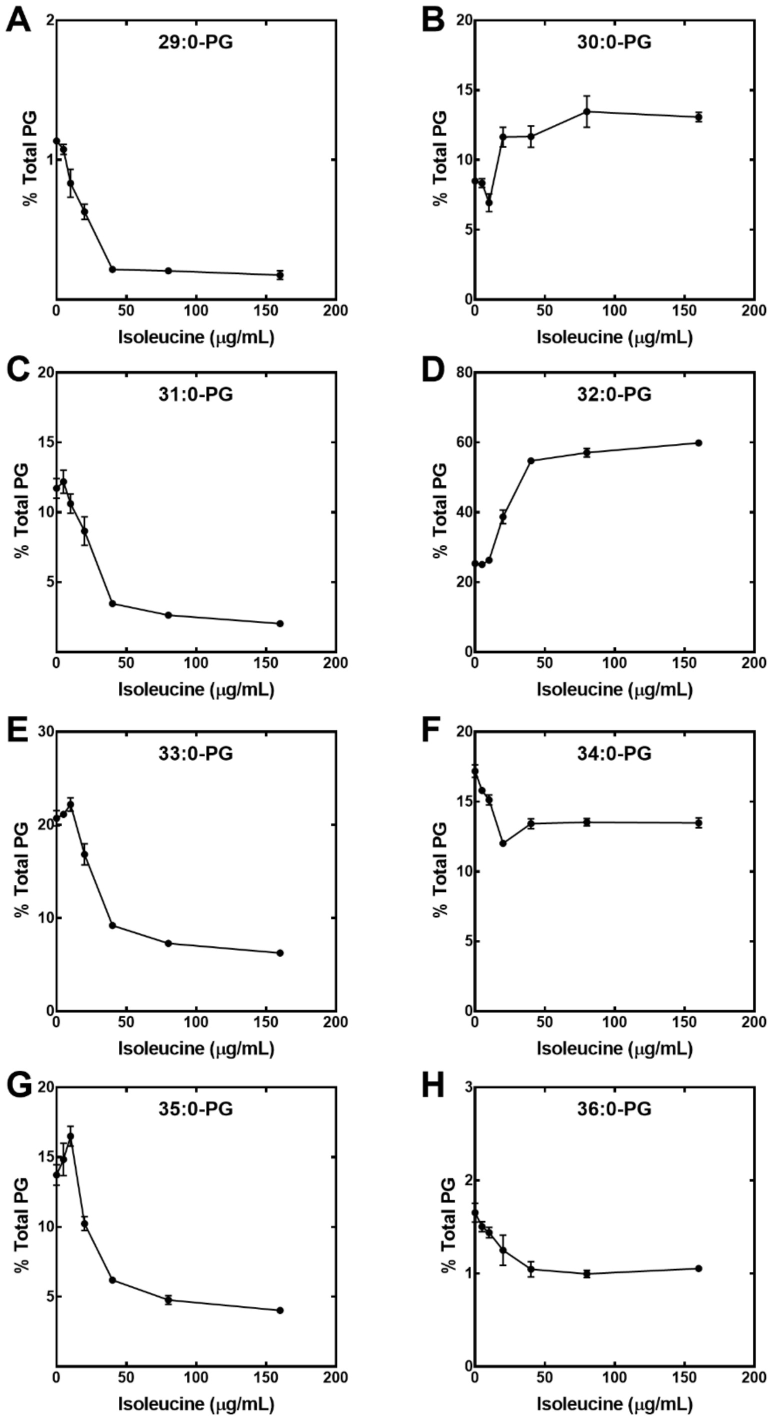

Figure S3. Ile control of PG molecular species composition. Strain AH1263 was grown in defined medium containing Ile concentrations between 0 and 160 μg/ml. Triplicate biological replicates were harvested at an A_{600} of 0.6, extracted and the PG molecular species distribution determined by LC-MS/MS. The contribution of each PG molecular species to the total was calculated using LipidView software (Sciex) and the contribution of each of the 8 species was plotted as a function of Ile concentration. *A*, 29:0-PG. *B*, 30:0-PG. *C*, 31:0-PG. *D*, 32:0-PG. *E*, 33:0-PG. *F*, 34:0-PG. *G*, 35:0-PG. *H*, 36:0-PG. The data are plotted as mean ± S.E.M. with three biological replicates.

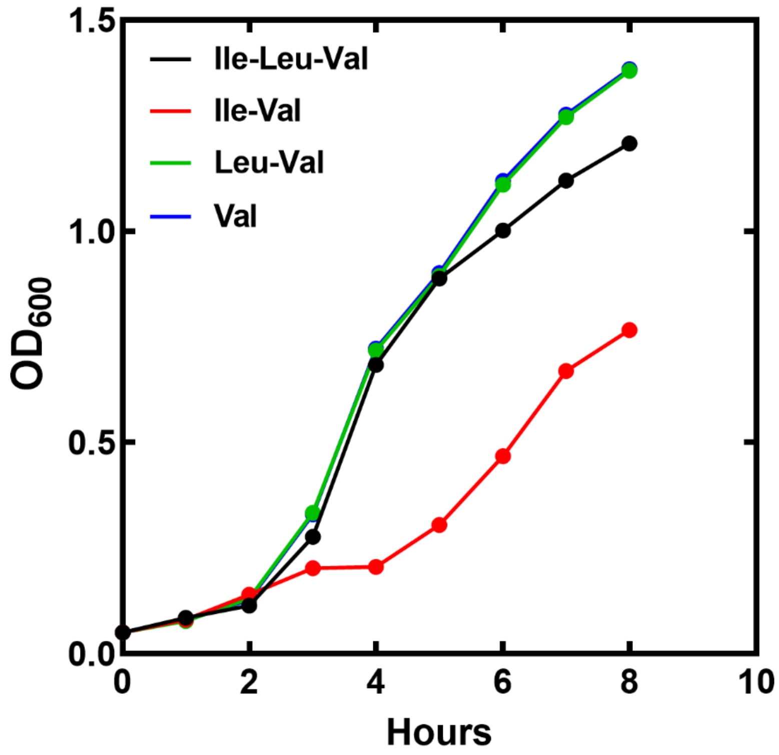

Figure S4. Branched-chain amino acid impact on the growth of *S. aureus* in defined medium. The growth rates of strain AH1263 in complete defined medium (black) containing Ile, Leu and Val (Table S2) is compared to the growth rate when one or more of the branched-chain amino acids were removed. Leu removed (red), Ile removed (green); and Ile + Leu removed (blue).
